# Single-Cell Transcriptional Analysis Reveals Novel Neuronal Phenotypes and Interaction Networks Involved in the Central Circadian Clock

**DOI:** 10.3389/fnins.2016.00481

**Published:** 2016-10-25

**Authors:** James Park, Haisun Zhu, Sean O'Sullivan, Babatunde A. Ogunnaike, David R. Weaver, James S. Schwaber, Rajanikanth Vadigepalli

**Affiliations:** ^1^Department of Pathology, Anatomy and Cell Biology, Daniel Baugh Institute for Functional Genomics and Computational Biology, Sidney Kimmel Medical College, Thomas Jefferson UniversityPhiladelphia, PA, USA; ^2^Department of Chemical and Biomolecular Engineering, University of DelawareNewark, NJ, USA; ^3^Department of Neurobiology, University of Massachusetts Medical SchoolWorcester, MA, USA

**Keywords:** single-cells, cell-network, transcriptional heterogeneity, transcriptional phenotypes, network topology

## Abstract

Single-cell heterogeneity confounds efforts to understand how a population of cells organizes into cellular networks that underlie tissue-level function. This complexity is prominent in the mammalian suprachiasmatic nucleus (SCN). Here, individual neurons exhibit a remarkable amount of asynchronous behavior and transcriptional heterogeneity. However, SCN neurons are able to generate precisely coordinated synaptic and molecular outputs that synchronize the body to a common circadian cycle by organizing into cellular networks. To understand this emergent cellular network property, it is important to reconcile single-neuron heterogeneity with network organization. In light of recent studies suggesting that transcriptionally heterogeneous cells organize into distinct cellular phenotypes, we characterized the transcriptional, spatial, and functional organization of 352 SCN neurons from mice experiencing phase-shifts in their circadian cycle. Using the community structure detection method and multivariate analytical techniques, we identified previously undescribed neuronal phenotypes that are likely to participate in regulatory networks with known SCN cell types. Based on the newly discovered neuronal phenotypes, we developed a data-driven neuronal network structure in which multiple cell types interact through known synaptic and paracrine signaling mechanisms. These results provide a basis from which to interpret the functional variability of SCN neurons and describe methodologies toward understanding how a population of heterogeneous single cells organizes into cellular networks that underlie tissue-level function.

## Introduction

The principal biological clock in mammals resides in the suprachiasmatic nucleus (SCN) of the hypothalamus and synchronizes circadian oscillations and behavioral rhythms throughout the body. Synchronization, which enables coordinated anticipation of the 24 h daily light/dark cycle, results from coherent, rhythmic output signals generated and adjusted by the SCN in response to photic inputs. This oscillatory behavior of the SCN arises from neurons, which exhibit autonomous circadian rhythms, interacting with one another via synaptic and paracrine signaling mechanisms to form cellular interaction networks that synchronize the oscillatory behavior of individual SCN neurons (Mohawk et al., 2012). Consequently, SCN tissue is able to generate synaptic and molecular signals that are more precise and rhythmic than those of individual neurons. Because synchronization results in part from emergent properties of the cell-interaction networks underlying SCN function, a better understanding of such properties requires knowledge of the functional behavior of these cellular networks and how their constituent components (i.e., single neurons) are organized.

Prior studies characterizing SCN neurons relied on intrinsic neurochemical features and spatial localization. For example, immunohistochemical (IHC) staining of neurons expressing vasoactive intestinal polypeptide (VIP), or arginine vasopressin (AVP) have shown that these neurons mainly localize within the ventrolateral (core) or dorsomedial (shell) regions of the SCN, respectively (Leak et al., 1999; Abrahamson and Moore, 2001; Morin, 2007). VIP+ and AVP+ neurons differ in the expression of cell–surface receptors and genes involved in circadian regulation (i.e., core clock genes) in response to light stimuli. Distinct spatial localization and transcriptional responses in VIP+ and AVP+ neurons have made these neuropeptides convenient neuronal phenotypic markers that have provided insight into the function and spatial organization of the photic input–oscillator–output system that entrains the SCN to a light/dark cycle (Gerkema et al., 1994; Jin et al., 1999; Van der Zee et al., 2002; Hamada et al., 2004).

However, single-cell level analyses have shown that individual neurons comprising SCN cell-networks are heterogeneous across multiple functional levels, as exemplified in single neuron firing patterns, which occur at different phases of the circadian cycle (Schaap et al., 2003), and in the period of oscillatory gene expression programs, which varies from 22 h to 30 h (Welsh et al., 1995; Karin et al., 1997; Ko et al., 2010). Additionally, while most VIP+ and AVP+ neurons exhibit intrinsic rhythmic firing rates and transcriptional programs, not all neurons (*in vitro*) exhibit circadian behavior. In the absence of synaptic signaling, these behaviors can become unstable, resulting in some neurons losing their intrinsic rhythmicity while others can spontaneously gain rhythmicity (Webb et al., 2009). Further, waves of gene expression travel through cultured SCN tissue in an orderly fashion (Evans et al., 2013) indicating that a complex phase relationship exists among neuronal oscillators, even when functionally coupled.

Understanding how neurons interact to form cell-interaction networks regulating circadian behavior is further confounded by inherent transcriptional heterogeneity in single neurons (Eberwine and Bartfai, 2011; Junker and van Oudenaarden, 2014). Our recent analysis of transcriptional states of single brainstem neurons suggests that this transcriptional heterogeneity may be understood in terms of synaptic and neuromodulatory inputs that drive neurons into distinct transcriptional states underlying neuronal function (Park et al., 2014a). Concomitantly, recent work has also shown that phase-shift behavior in the SCN arises from multi-genic networks (Araki et al., 2006; Hastings et al., 2008; Porterfield and Mintz, 2009; Zhu et al., 2012). These results, when considered with the input-driven nature of photosensitive SCN neurons and the region-specific peptide expression behavior of neurons throughout the SCN, suggest that analyzing both the transcriptional responses of individual SCN neurons to photic inputs and their spatial distribution throughout the nucleus would provide insight into the neuronal phenotypic states and organization of these states comprising the cell-networks that drive the robust and synchronized outputs of this brain nucleus.

In this study, we aimed to reconcile the heterogeneous behavior of individual SCN neurons with the coordinated behavior of the SCN by characterizing the transcriptional and spatial diversity of these neurons under light-induced phase-shifting behavior and developing a neuronal network model with which to interpret the single-cell heterogeneity in the context of tissue-level function (Eberwine et al., 2013). Therefore, we sampled hundreds of individual SCN neurons while tracking their position *in situ* using laser capture microdissection (Espina et al., 2006; Macdonald et al., 2008; Park et al., 2014a; Chung and Shen, 2015; Datta et al., 2015). We subsequently characterized these neurons, independent of prior knowledge of known SCN neuron-types, using their transcriptional states across a panel of circadian-related genes in order to characterize neurons more comprehensively than might be possible from a single biomarker or a select few biomarkers (Usoskin et al., 2014). Given the previous extensive characterization of the transcriptional regulation of circadian rhythms (Sato et al., 2003; Araki et al., 2006; Porterfield et al., 2007; Doherty and Kay, 2010), we focused on measuring expression levels of 96 genes relevant to intercellular, paracrine signaling and gene expression programs involved in neuronal phase-shifting behavior (Araki et al., 2006; Hastings et al., 2008; Porterfield and Mintz, 2009; Zhu et al., 2012). Prior studies have shown that gene panels of similar scale and functional diversity provide a sufficient basis to define a framework within which to interpret the transcriptional heterogeneity of single cells in the brain (Park et al., 2014a; Darmanis et al., 2015). We collected single SCN neurons from dark-adapted mice or mice experiencing a light-induced (light-pulsed) phase shift in their circadian rhythms. Using multivariate analytical techniques and a particular technique from the field of graph network theory known as community structure detection, we analyzed the transcriptional states of these neurons and identified a molecular organizational framework within which distinct functional groups of SCN neurons function and interact.

## Materials and methods

### Animals

C57BL/6J male mice between 4 and 6 weeks old purchased from Charles River Laboratories (Wilmington, MA) were housed within 12 h light, 12 h dark cycles and given free access to food and water. Warm white fluorescent bulbs (150 lux) were used for the light cycle. After 10 days of light/dark cycle entrainment, the lights were switched off. On the second day of the constant dark period, animals were given a 1 h light exposure (150 lux of white light) at Zeitgeber time (ZT) 14, 2 h into their subjective dark period, and sacrificed 1 h later at ZT 15 (light-pulsed). SCNs were also collected from non-light-pulsed animals at ZT 15 (dark adapted). Animals were euthanized by carbon dioxide asphyxiation in dim red light, and brains were extracted in light. Hypothalamic tissue blocks were dissected and embedded in Optimal Cutting Temperature (OCT) embedding medium and frozen on dry ice. OCT-embedded tissue samples were stored at −80 C prior to sectioning and laser capture microdissection. All protocols were approved by the TJU Institutional Animal Care and Use Committee.

### Staining and histological analysis

In order to minimize RNA degradation and maintain quality of the tissue samples for laser capture microdissection we developed a rapid immunofluorescent staining protocol. OCT-embedded hypothalamus blocks were sectioned in a cryostat into 10 um thick sections and thaw-mounted onto glass slides. Sections were fixed in 75% ethanol for 30 s, then blocked and permeabilized with PBS containing 2% BSA (Sigma-Aldrich) for 30 s. Afterwards, sections were incubated with the primary antibody anti-NeuN 1:25 (Millipore®). PBS-washed slides were incubated in the dark for 3 min at room temperature with the secondary antibody Alexa-488 anti-mouse 1:50 diluted in PBS containing 2% BSA. The slides, again washed with PBS, subsequently underwent a standard dehydration process (75% ethanol, 30 s; 95% ethanol, 30 s; 100% ethanol, 30 s; 100% ethanol, 30 s), rinsed briefly in Xylenes (Sigma-Aldrich) for 1 min, and then transferred into another bath of fresh xylenes for 5 min to further remove any trace of ethanol. Finally, the slides were air-dried for 5 min prior to laser capture microdissection.

### Single cell sampling and high-throughput qRT-PCR

We collected 352 single SCN neurons from the light-perturbed or dark-adapted mice using laser capture microdissection (LCM, Espina et al., 2006). Neurons throughout the SCN were selected in an unbiased fashion and their anatomic location recorded. Within each coronal section collected, beginning with the first appearance of the rostral SCN, spatial coordinates within a 10 μm × 10 μm grid system were recorded to determine neuron location in each coronal section. To indicate the grid location, two sets of seven divisions were used, one set beginning from brain midline extending laterally and the other beginning from the ventral SCN border with the optic chiasm extending dorsally. Seven divisions were used along each axis to indicate the grid location.

### cDNA preparation and high-throughput quantitative real-time qPCR

A standard reverse transcription protocol using random primers generated cDNA from RNA samples. The cDNA was then pre-amplified with a pool of 96 pairs of PCR primers using TaqMan PreAmp Master Mix (Applied Biosystems, Foster City, CA). All cDNA samples underwent 20 cycles.

The expression level of 96 genes was measured using a high-throughput quantitative PCR platform (BioMark™, Fluidigm^©^) according to the manufacturer's instructions. The same primer set used in the pre-amplification process was the basis for the probe-based quantitative PCR. The 96.96 dynamic gene expression arrays were used. qPCR was based on the Universal Probe Library (Roche, Indianapolis, IN). A detailed list of probe and primer sets is included in the supplemental (Table S3). These gene/primer pairs were pre-validated by both standard PCR and qPCR analysis using cDNA generated from mouse hypothalamic RNA (TakaRa^©^, Mountain View, CA).

### Data normalization

Expression levels were normalized by using the −ΔΔC_t_ method (Spurgeon et al., 2008) in a two-step process. Briefly, gene expression within a single neuron was first normalized relative to the average of housekeeping genes (*Actb, Hprt, Atp5b*) selected by using previously developed gene-stability evaluations across measured samples (Vandesompele et al., 2002), a step resulting in a −ΔC_t_ value. Next, −ΔC_t_ values within genes were median-centered (across both dark-adapted and light-treated samples) resulting in a −ΔΔC_t_ value that allowed the relative gene expression values to be compared across both treatment groups. Modified *z*-values were then calculated by means of dividing the −ΔΔC_t_ the standard deviation within a gene assay across all single-cell samples, values that were used for data visualization using heat maps. Reference the Supplemental Information for additional information and quality control assessment of qRT-PCR data.

### Silhouette score

This method quantifies the consistency across members within a designated cluster. A silhouette score is calculated for each member within a cluster and then an average score is used to represent how similar members are within a cluster. Let *a*(*i*) represent the average dissimilarity of member *i* relative to all other members within its designated cluster, the lower the value of *a*(*i*), the better the cluster assignment. Average dissimilarity values of member *i* relative to all other clusters are then calculated, from which the lowest average dissimilarity value is chosen as *b*(*i*). Using the values *a*(*i*) and *b*(*i*), we then calculate the silhouette score *s*(*i*) as follows:

$$s\left(i\right)=\frac{b(i)-a(i)}{\max\{a\left(i\right),b\left(i\right)\}}$$

From the above equation, silhouette scores fall within a range of −1 ≤ *s*(*i*) ≤ 1

A value approaching 1 indicates that the difference of a member across a distinct cluster is much larger than the difference within its designated cluster meaning that the member is well matched and vice-versa. In order to quantify the overall similarity across members within a cluster, we then calculate the arithmetic average of all *s(i)* values within a group. Similar to an individual score, an average score approaching 1 indicates a well-defined cluster, –1 indicates an ill-defined cluster, and 0 represents a neutral clustering of members. Silhouette scores were determined using the silhouette function provided in the R package “cluster” (Maechler et al., 2012).

### Community structure detection

Community structure detection based on the leading eigenvector of the community matrix is a graphical analysis approach developed by Newman (2006) that is used to analyze graphical networks, consisting of nodes and edges. This technique is used to identify community structures or modules of highly interconnected nodes hidden in an existing network topology. Here, a network graph is represented as a community (i.e., modular) matrix, which represents the difference between the actual number of edges connecting to a node and the expected number of edges connecting to a node by chance. This modular matrix represents the degree to which a node belongs to a highly interconnected module (as opposed to belonging to a module by random chance). The modular matrix is then partitioned into a set of representative eigenvectors (of node members) that make principal contributions to the modularity of the original network topology (Newman, 2006). Conceptually similar to PCA, community detection seeks to identify groups of highly interconnected nodes that principally contribute to the overall modularity of the original network graph. Community structure detection was performed using the leading eigenvector community function provided in the R package “igraph” (Csardi and Nepusz, 2006).

## Results

**Figure 1:**
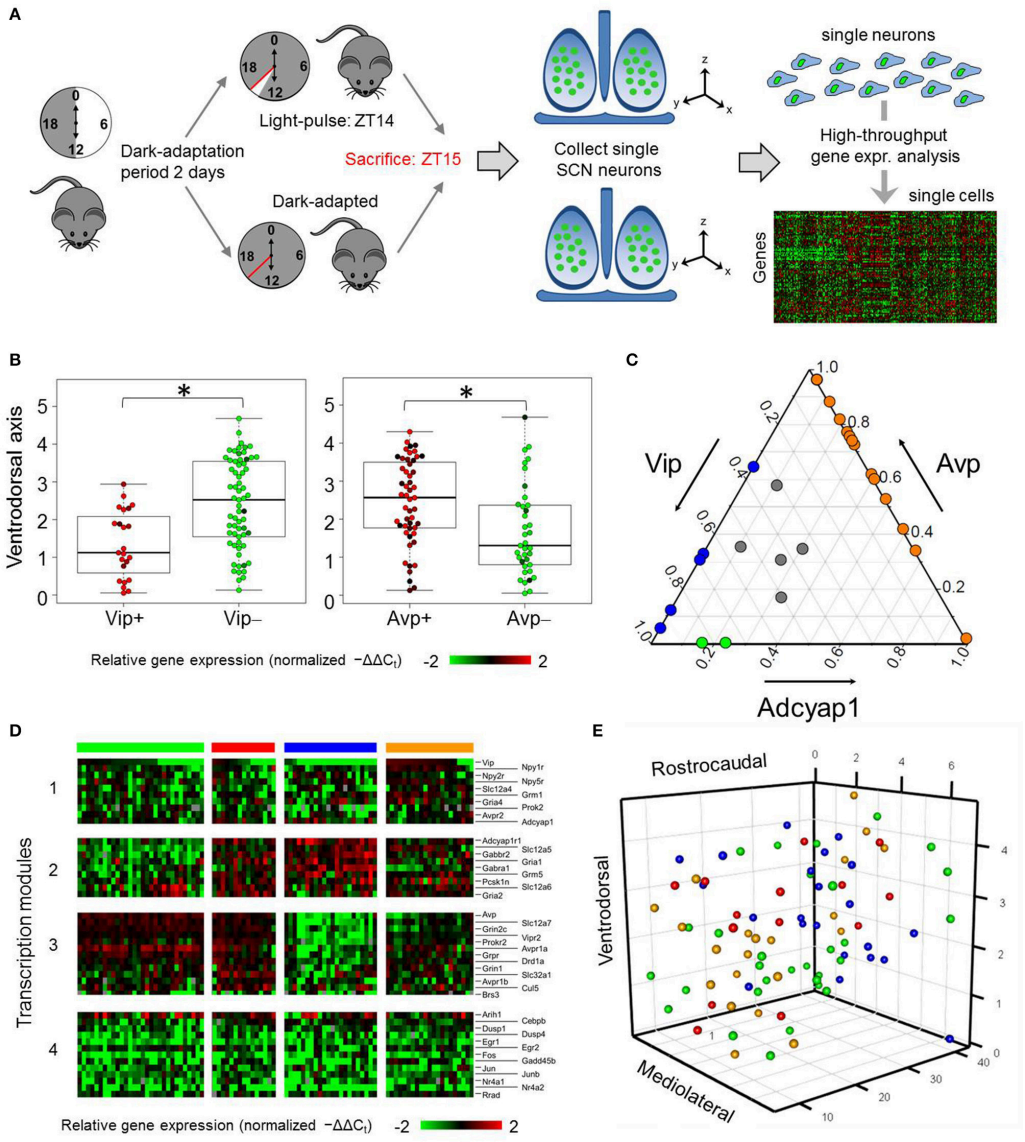
**Single-cell analysis of SCN neurons from dark-adapted mice reveals multiple states. (A)** Process flow diagram of experimental approach taken to obtain single-neuron samples and transcriptomic measures from mice that adapted to a dark-dark (DD) cycle or light pulse (LP) treatment. **(B)** Ventrodorsal positioning of individual *Vip*+ (−ΔΔC_t_ > 0) and *Vip*- (−ΔΔC_t_ ≤ 0) neurons. A Wilcoxon rank sum test was performed to determine if there were significant differences in ventrodorsal positioning between the two neuron groups. A *p*-value of 1.78 × 10^−4^ indicates that the null hypothesis can be rejected and that there is a statistically significant difference between the ventrodorsal positioning of *Vip*+ and *Vip*- neurons. Similarly, significant differences in ventrodorsal positioning were observed between *Avp*+ and *Avp*- neurons (Wilcoxon rank sum test *p*-value = 1.14 × 10^−3^). ^*^indicates statistically significant differences (*p* < 0.05). **(C)** Ternary plot of the proportional expression levels of the neuropeptide genes *Vip, Avp*, and *Adcyap1*, which sum to a total value of 1. Each circle represents an individual neuron's compositional *Vip-Avp-Adcyap1* expression profile. These samples form a subset of single neurons from dark-adapted mice that have positive normalized expression of the three genes of interest. Colored circles represent individual neurons with positive coexpression of *Vip/Avp* (blue), *Vip/Adcyap1* (green), *Avp/Adcyap1* (orange), and *Vip/Avp/Adcyap1* (gray). **(D)** Heat map visualizes relative expression levels (−ΔΔC_t_) of 32 key neuropeptide, receptor, and light-response genes measured in the 88 single neurons from DD mice. Hierarchical clustering, based on the Pearson correlation coefficient, revealed four groups of genes (transcription modules) showing correlated expression across four neuronal subpopulations (groups of columns) defined by gene expression profiles. Colored bars above each group correspond to specific subpopulations. **(E)** Mapping of SCN neuronal subpopulations. Color annotation of subpopulations identical to gene expression-based groups defined in **(D)**. Single neurons were plotted based on their recorded spatial coordinates (*Material and Methods*) along the mediolateral, ventrodorsal, rostrocaudal axes of the SCN.

We collected 352 single SCN neurons from mice kept either in constant darkness for 2 days (dark-dark or DD mice, *n* = 2) or kept in darkness for 2 days and then exposed to a light-pulse (LP) at a clock time corresponding to 2 h after lights-out of the previous 12 h light-dark cycle [Zeitgeber time (ZT) 14; LP mice *n* = 6]. Brains were collected after 1 h of light exposure in the LP group and at the corresponding clock time in the DD group (ZT15, Figure 1A). Single-cell cDNA was analyzed with 30,624 individual qRT-PCR measurements. Numerous stringent quality control tests were used to assess the data and ensure that only high-quality single-cell data was included in the subsequent analysis. Of the total single-neuron samples collected, 29 samples were excluded due to failed reactions, improper sample loading, or poor signal quality. Ultimately, 88 neurons from DD mice and 235 from LP mice were analyzed. We measured expression profiles using a high–throughput qPCR platform (BioMark™, Fluidigm^©^), which measures gene expression over five orders of magnitude with minimal technical (Figure S1) variability indicating that gene expression variation measured is biological rather than technical in nature (Park et al., 2014a). Measurements included transcripts involved in intracellular signaling pathways, regulated downstream targets, and core-clock functions (Table S1). Sample collection and measurements were performed under established procedures that minimize cross–contamination of samples (Wang et al., 2002; Ye et al., 2003; Zhang et al., 2003; Espina et al., 2006; Macdonald et al., 2008; Park et al., 2014a). Since expression levels were normalized to the mean expression level of *Actb, Atp5b*, and *Hprt*, three housekeeping genes that do not exhibit circadian expression rhythms (Balsalobre et al., 1998; Gossan et al., 2013; Westfall et al., 2013; Cleal et al., 2014), and six assays were excluded due to poor signal quality or assay contamination, neurons were characterized based on the normalized expression of 87 genes. Moreover, given the variation observed in our single-neuron transcriptional dataset, statistical analysis was performed on multiple permutations of the dataset to verify the significance of the pairwise gene and cell correlation coefficient thresholds used to identify the molecular signatures and organizational framework described herein (Figures S12,S13).

### Dark-adapted neurons exhibit multiple functional states

Our analysis revealed substantial transcriptional heterogeneity across the DD neurons, evidenced not only in the wide range of expression levels of neuropeptide and membrane receptor genes but also in the combinations of several key neuropeptide genes expressed. For example, *Vip* showed binary-like expression across DD neurons, of which 45% expressed *Vip* levels below the detection limit. Single neurons expressing *Vip* were localized within the ventral portion of the SCN (Figure 1B), as expected (Abrahamson and Moore, 2001; Moore et al., 2002; Mohawk et al., 2012). *Avp* expression, however, spanned a 4000-fold expression range across the DD neurons. Surprisingly, gene expression of adenylate cyclase-activating polypeptide (*Adcyap1*), which codes for the neuropeptide PACAP [a molecular input signal generated by primary ganglion neurons in the retinohypothalamic tract (RHT), which innervates the ventral regions of the SCN] occurred in approximately 25% of neurons sampled from DD mice (Figures 1C,D). Contrary to previous results, which defined distinct SCN neuronal populations based on their exclusive production of VIP or AVP (Webb et al., 2009; Welsh et al., 2010), many DD neurons sampled expressed combinations of *Vip, Avp*, and *Adcyap1* (Figure 1C) suggesting that these neurons may exist in various functional states.

Subsequent hierarchical clustering analysis based on 32 neuropeptide and membrane receptor genes, as well as 13 light-response genes, performed on the DD neurons revealed four neuronal subpopulations having distinct expression profiles (Figure 1D, Figure S2). In addition to co-expressing combinations of *Vip, Avp*, and *Adcyap1*, these subpopulations were characterized by correlated expression patterns of three distinct groups of genes (i.e., transcription modules; Figure 1D, Table S4) with Pearson correlation coefficient values greater than the statistically significant threshold (Figure S3). Sample clusters of DD neurons were also supported by the presence of neuron sample clusters detected from a principal component analysis (PCA) of these neurons (Figure S4). Despite showing no distinct expression patterns across the neuronal subpopulations, light-response genes including *Fos, Egr1, Egr2, Jun*, and *Junb*, forming a fourth transcription module, were included in Figure 1D to serve as a qualitative internal validation of our experimental approach and analysis. Because SCN neurons increase expression of these genes upon light-mediated activation (Araki et al., 2006; Porterfield et al., 2007; Porterfield and Mintz, 2009), we expected downregulated expression of these genes in DD neurons, which was what was observed (Figure 1D). The remaining genes measured did not lead to further distinction of subpopulations and were therefore not included in the heat map, (refer to Figure S2 for a heat map including all genes). While correlations between gene expression states related DD neurons to a particular subpopulation, there was no clear spatial organization among these subpopulations (Figure 1E).

### Gene expression profiles distinguish dark-adapted from light-pulsed SCN neurons

**Figure 2:**
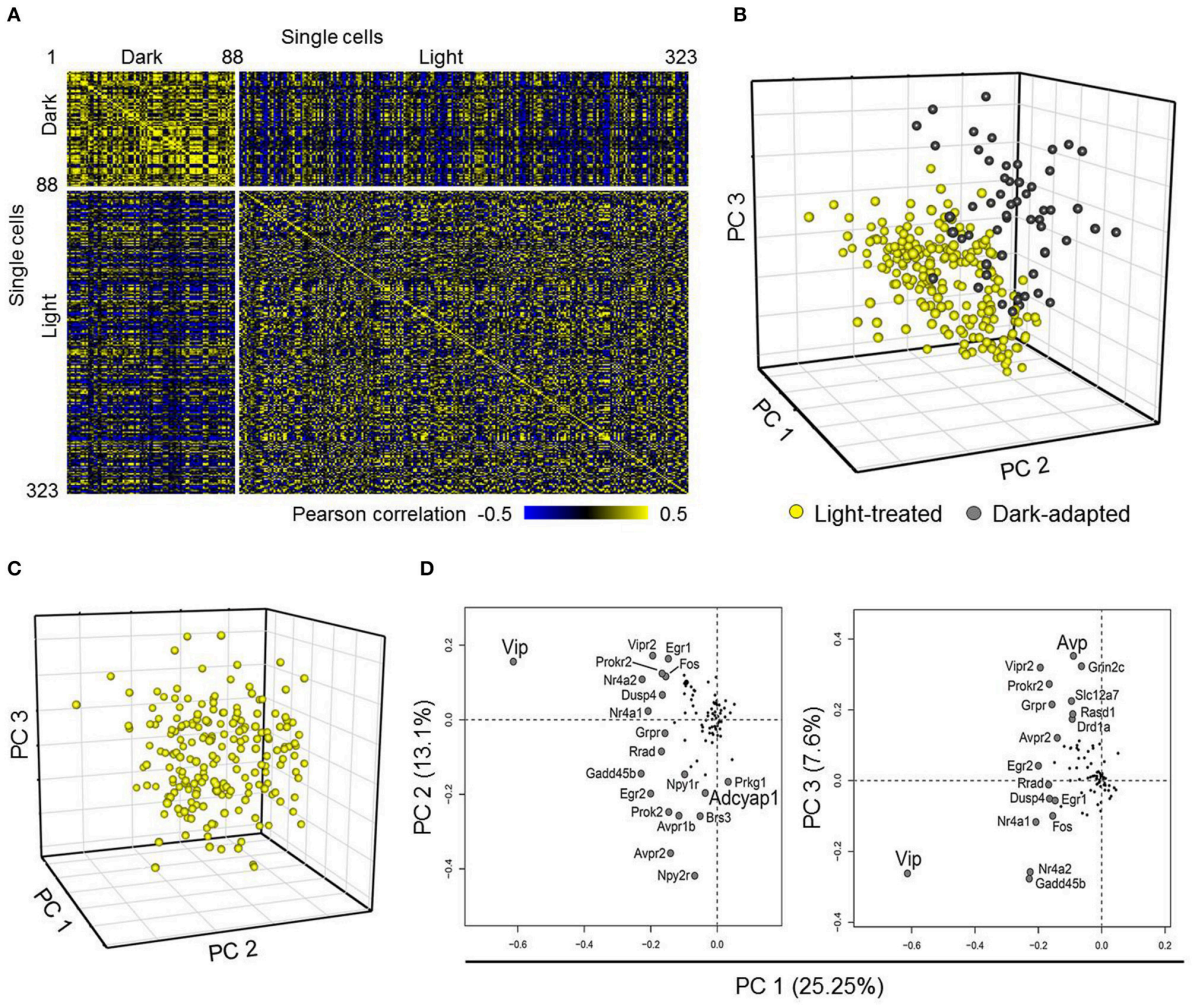
**Comparing neurons from dark-adapted and light-treated mice. (A)** Pearson correlation of 332 single neurons collected across dark-adapted (88 neurons) and light-treated (235 neurons) mice. **(B)** Scores plot obtained from a Principal Component Analysis (PCA) on the single neurons (spheres) sampled shows that neurons responding to the light pulse (yellow) form a distinct cluster from that of the dark-adapted neurons (dark-gray) along the first three principal components. The distinct clusters indicate distinct transcriptional states between these two treatment groups. **(C)** Scores plot obtained from PCA of only neurons from light pulsed animals shows a large amount of variability as indicated by the large spread of neurons. **(D)** Loading values obtained from the PCA analysis of neurons taken from light-perturbed animals are plotted for PC1 and PC2 and PC1 and PC3. The labeled genes contribute more to the observed variability in the data, with the signaling neuropeptide genes *Vip* and *Avp* having the largest contributions to neuronal variability along PC1 and PC2. *Adcyap1* and other labeled genes contribute to variability along PC3.

Concomitantly, we compared the transcriptional states of the DD and LP neurons using several multivariate analytical techniques to verify light-mediated changes in neuronal state. The Pearson correlation coefficient was used as a measure of similarity between all possible pairs of transcriptional profiles of neurons, which we visualized in a heat map. This analysis revealed two distinct clusters composed predominantly of positive correlation coefficients among pairs of neurons within treatment groups indicating that transcriptional states were more similar within than across treatments (Figure 2A). In a separate analysis, we used PCA to characterize the variation across the transcriptional states of DD and LP neurons by projecting the multi-genic expression states into a lower-dimensional gene expression space defined by new coordinate axes (i.e., principal components). These components are ordered in such a way that variation in the data is captured along the components in a successively decreasing manner. We found that the first three principal components retained 46% of the variation in the original data, which was sufficient to distinguish neuronal states between the two treatment groups (Figure 2B).

Next, we examined the weighted contributions (i.e., loadings) each gene had to each of the first three principal components to determine which genes had the largest influence on the distribution of transcriptional states of LP and DD neurons along these principal components. Genes having the largest influences (i.e., loadings with the largest magnitude values) along the first three principal components included light-induced genes such as *Per1, Egr1, Egr2, Fos*, and *Jun* (Shigeyoshi et al., 1997; Dardente et al., 2002; Yan and Silver, 2002), and GABA-associated inhibitory signaling genes such as *Gabra1* and *Slc12a5*, which are involved in phase-shifting responses in the SCN and synchronizing SCN neurons (Liu and Reppert, 2000; Allen et al., 2015). These results further support the idea that single-cell transcriptional profiles can be used to distinguish distinct functional states of neurons between treatment groups, as previously reported (Durruthy-Durruthy et al., 2014; Park et al., 2014a; Llorens-Bobadilla et al., 2015).

Although the transcriptional states of neurons between the two treatment groups were distinct, the large spread of transcriptional states of the LP neurons in the principal component space (PC 1–3) indicated that a large amount of variation within the LP neuronal dataset remained (Figure 2B yellow spheres). A PCA performed on the LP neurons indicated that variation across the transcriptional states of these neurons were due in part to expression variations of *Vip, Avp*, and *Adcyap1* (Figures 2C,D). Interestingly, *Adcyap1* expression was observed across multiple neurons throughout the SCN under both treatment conditions (Figure S5), suggesting that endogenous PACAP production, in addition to light-induced production in the RHT, may also play a role in synchronization. As *Vip, Avp*, and *Adcyap1* are involved in circadian regulation and were some of the major contributors to gene expression variation in the LP neurons (Figure 2D), we examined how well a biased classification approach, one based on the expression levels of these three genes, would be able to categorize transcriptional phenotypes of SCN neurons.

### Neuropeptide-based classification poorly characterizes transcriptional states of light-pulsed neurons

**Figure 3:**
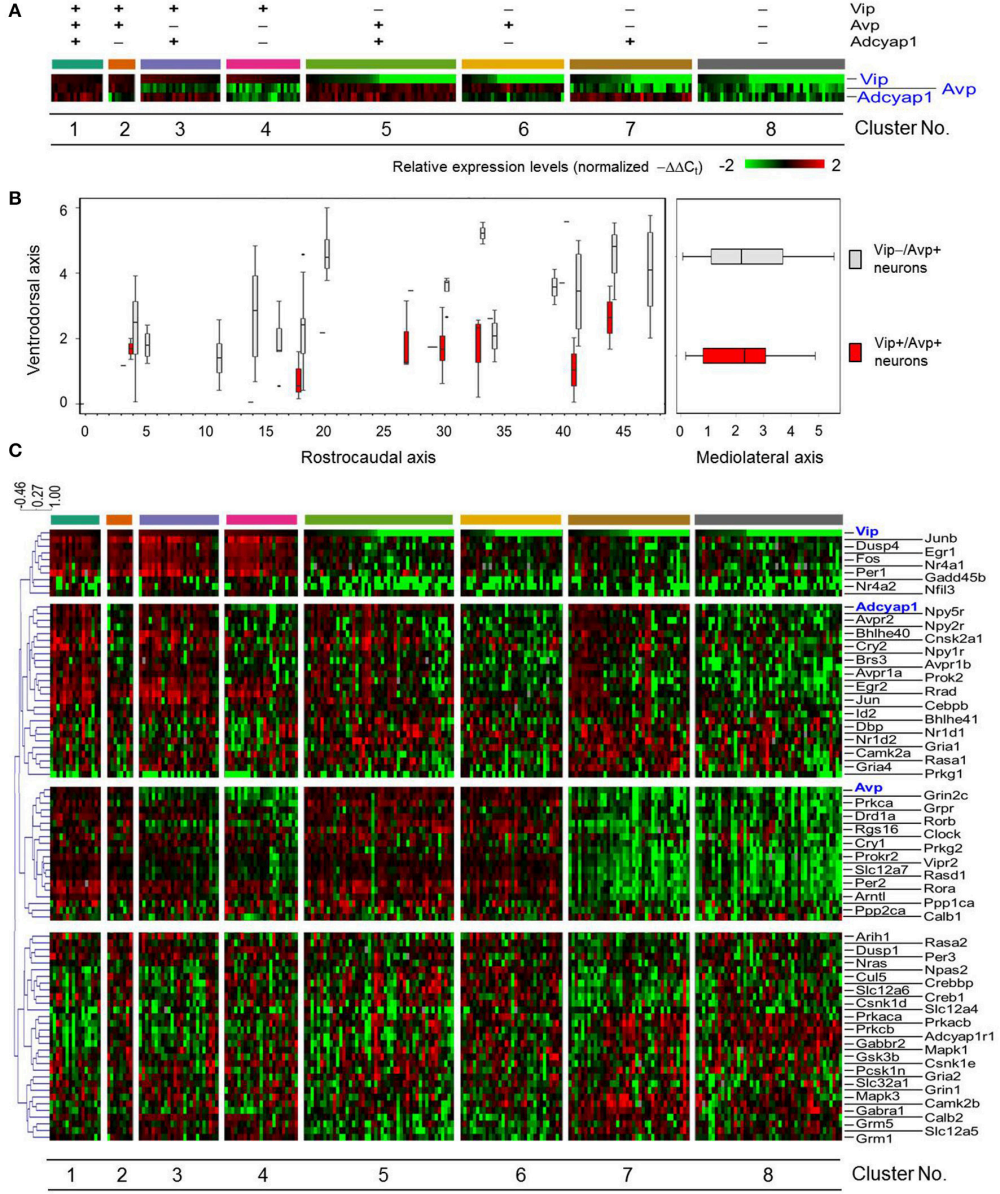
**Sorted gene expression profiles of SCN neurons in light-perturbed mice with respect to *Vip*, *Avp*, and *Adcyap1*. (A)** Heat map visualizing normalized gene expression of *Vip, Avp*, and *Adcyap1* across light-pulsed (LP) neurons. Neurons are categorized based on positive (−ΔΔC_t, *i-gene*_ > median (−ΔΔC_t, **gene**_) or negative (−ΔΔC_t, *i-gene*_ ≤ median (−ΔΔC_t, **gene**_) expression. Gene expression levels were normalized per method described in *Materials and Methods*. **(B)** Boxplots show distribution of *Vip*+/*Avp*+ neurons and remaining sampled neurons along ventrodorsal, mediolateral, and rostrocaudal axis of SCN. The boxplots on the left show that the majority of *Vip–/Avp*+ neurons are positioned dorsally relative to the *Vip*+*/Avp*+ neurons. **(C)** Heat map visualizing expression of 87 genes across light-treated neurons. Neurons are organized as in **(A)**. Dendrogram indicates how genes were grouped was based on Pearson correlation coefficient.

Classifying neurons based on a binary classification of expression levels for *Vip, Avp*, and *Adcyap1*, with neurons either demonstrating positive (−ΔΔC_t_ > median gene expression) or negative expression (−ΔΔC_t_ ≤ median gene expression), yielded eight clusters (Figure 3A). Similar to the behavior of DD neurons, a small subset of LP neurons (10%) co-expressed *Vip, Avp*, and *Adcyap1* (Figure 3B), which aligns with prior observations of *Vip* and *Avp* co-expression reported by Romijn et al. (1997) and Mieda et al. (2015).

**Figure 4:**
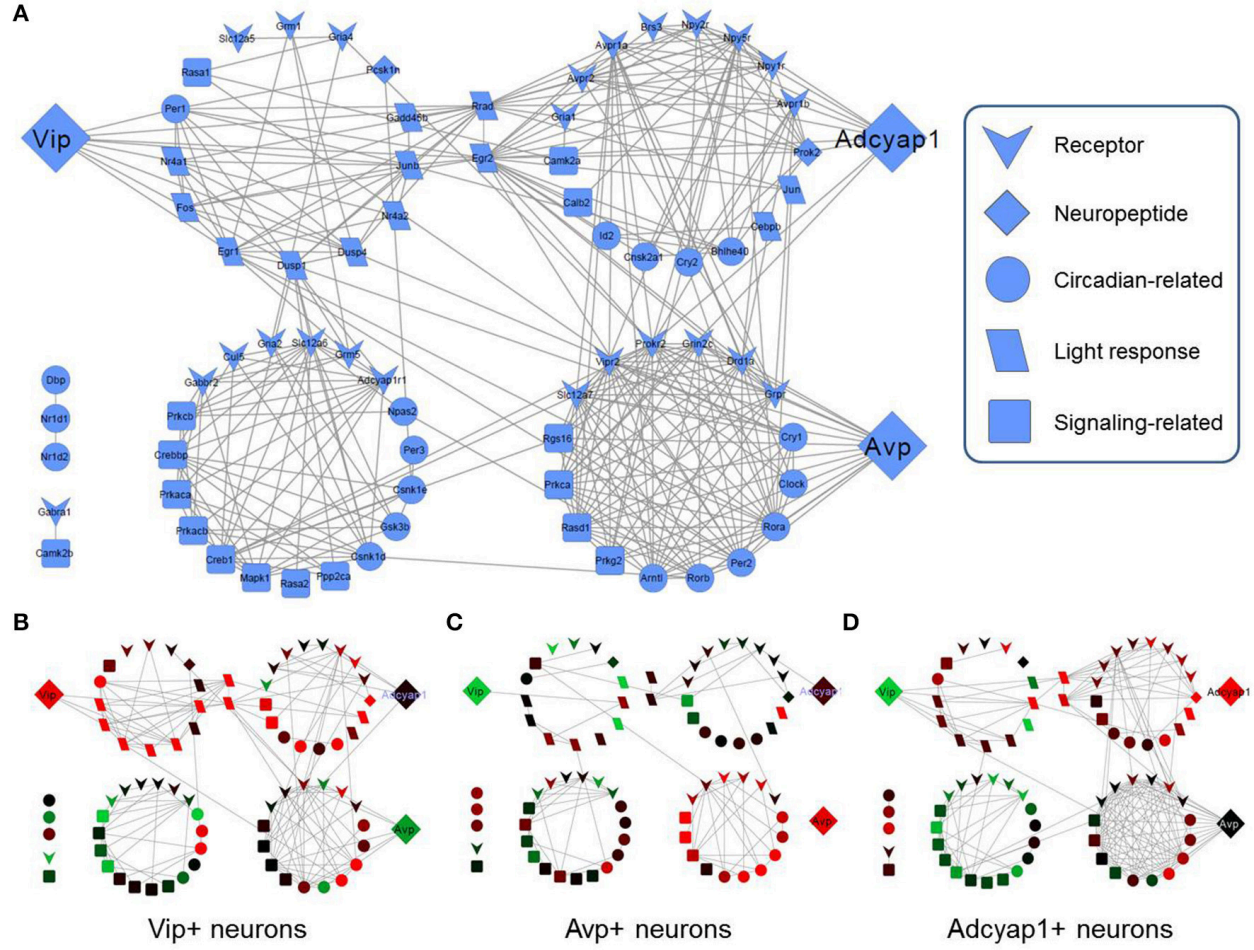
**Gene correlation networks within specific SCN cell-types. (A)** Combined gene correlation network across all genes and all subsets of neurons. Edges between nodes (genes) correspond to a Pearson correlation coefficient greater than 0.5, which was determined to be a statistically significant threshold (Figure S6). *Vip, Avp*, and *Adcyap1* are separated from their highly interconnected gene clusters in order to highlight these specific neuropeptide genes. Node placement is identical in the subsequent correlation networks for specific neuron-types (B–D). **(B)** *Vip*+ neuron gene correlation networks. When *Vip* expression is upregulated, few correlations are shared with other genes whereas *Vip2r* shows a larger number of gene correlations when it is expressed at high levels. **(C)** *Avp*+ neuron gene correlation network. Similar correlation behavior among genes with respect to neuropeptides and receptors is observed. Moreover, minimal correlations are observed among genes with the receptors for *Avp* (*Avpr1a, Avpr2a*) in *Avp*+ neurons. **(D)** *Adcyap1*+ neuron gene correlation network. Additional gene correlation networks for additional subsets of neurons are included in the Supplement (Figures S5–S9). Gene correlation networks were constructed using Cytoscape® version 2.8.4.

A heat map of the rearranged multi-genic profiles of LP neurons revealed four transcription modules that showed distinct, correlated expression patterns across several, but not all of the eight neuronal groups (Figure 3C). Since gene expression within the transcription modules appeared to correlate with *Vip, Avp*, and *Adcyap1* expression, we expected that these genes would act as central regulators or hubs in gene networks where the expression of core clock and other functional genes would be co-regulated. We subsequently identified statistically significant correlative relationships (Pearson correlation coefficient >0.5, Figure S6) among all pairwise combinations of the 87 genes measured and developed gene correlation networks to investigate gene regulatory network behavior (Park et al., 2014b; Moignard et al., 2015; Stegle et al., 2015). Gene correlation networks were developed from expression data across LP neurons and subsets of LP neurons, defined by positive normalized expression of key neuropeptide (*Vip*+, *Avp*+, *Adcyap1*+ [Figures 4B–D]) and receptor genes (Figures S7–S9) involved in circadian rhythmicity and synchronization. The correlation network across all LP neurons showed a large number of pairwise correlations involving *Vip, Avp*, and *Adcyap1* (Figure 4A).

However, correlation networks within the subsets of LP neurons revealed few statistically significant correlations involving these three neuropeptide genes. Within *Vip*+ LP neurons, *Vip* shared correlations with only three other genes (Figure 4B). Similarly, neither *Adcyap1* nor *Avp* shared any pairwise correlations in *Adcyap1*+ or A*vp*+ LP neurons, respectively (Figures 4C,D). To further verify the ability (or inability) of this neuropeptide expression-based categorization to describe the transcriptional states of SCN neurons, we assessed both qualitatively and quantitatively how well the transcriptional states of these neurons clustered with respect to this categorization scheme. Multidimensional scaling (MDS, Supplemental Information), hierarchical clustering, and minimum spanning trees (Figure S10) showed repeatedly poor consistency across the transcriptional states within the eight clusters. Furthermore, nearly all silhouette scores (Figure S10 legend), a quantitative measure of the similarity of transcriptional states within each group (Materials and Methods), were negative, indicating poor consistency across transcriptional states within these groups. These results suggest that despite their utility in defining SCN neuron-types, categorization of transcriptional states based on *Vip, Avp, Adcayp1* expression is not comprehensive enough to describe the single-neuron heterogeneity observed.

### Distinct single-cell transcriptional phenotypes in light-pulsed neurons

**Figure 5:**
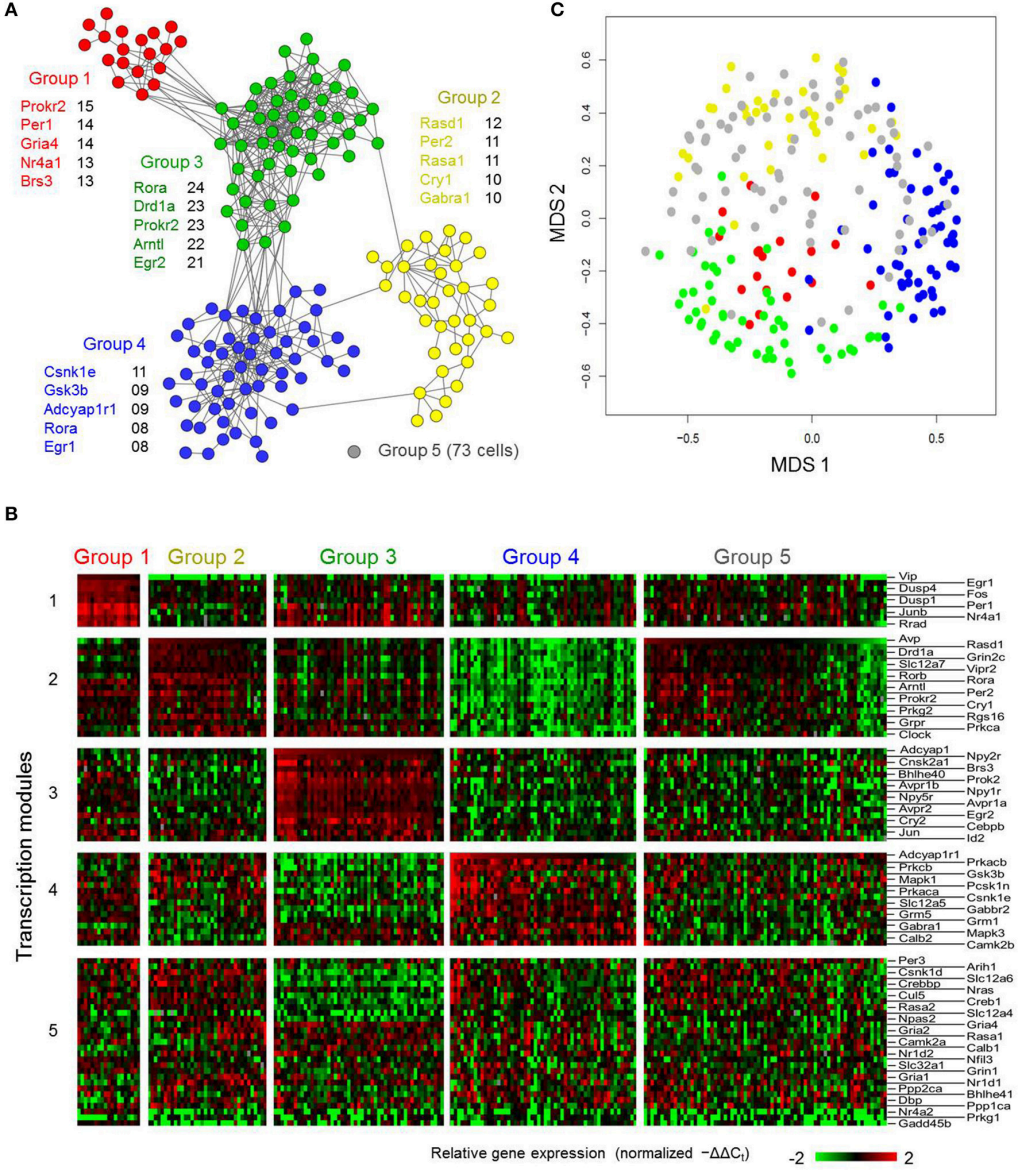
**High-dimensional gene expression analysis reveals novel SCN cell-types. (A)** Neuron correlation network based on Pearson correlation analysis across the multi-genic (87 genes) transcriptional states of the light-pulsed neurons. Each node represents an individual light-treated neuron. Edges indicate positive Pearson correlation coefficients >0.5. Highly interconnected groups of neurons, representing community structures within the correlation network, are labeled and colored accordingly. A fifth group of neurons (representative gray node) showed little or no correlations with other neurons. Genes listed next to each group correspond to the five genes with the highest number of correlations with the other genes (across cells) within the defined neuronal group. **(B)** Heat map visualizing the underlying transcriptional states corresponding to the SCN neuronal subtypes identified in **(A)**. **(C)** MDS 2-dimensional plot of light-treated neurons, similar to Figure S10C, with new neuronal group annotation **(A)**. Using this new neuronal group annotation, distinct clusters are apparent, with the exception of Group 5 neurons that are scattered throughout the plot which reflects some of the similarities that these neurons share transcriptionally to the neurons of the other groups. Silhouette scores for the neuronal groups were calculated to be: Group 1 (0.554), Group 2 (0.239), Group 3 (0.321), Group 4 (0.324), Group 5 (−0.258).

Due to the poor consistency of transcriptional states within clusters defined in the earlier approach, we sought an alternative way to characterize this transcriptional heterogeneity. Therefore, we applied an approach that relied on the full extent of the multi-genic transcriptional states measured to group the LP neurons. Neurons sharing similar transcriptional states (neuron-pairwise Pearson correlation coefficient ≥0.5, empirically determined to be statistically significant threshold [Figure S11]), were assumed to exist in similar functional states and to form distinct neuronal phenotypes. We constructed a neuronal correlation network by connecting LP neurons having similar transcriptional states. Subsequently we performed a topological analysis (Clauset et al., 2004; Newman, 2006) on the neuronal correlation network to identify highly interconnected modules within the network that were representative of neuronal phenotypes. We identified four highly interconnected modules or neuronal groups and created a fifth group that consisted of neurons showing a minimal number of or no significant correlations with any other neurons (Figure 5, gray node). A reorganized heat map of the transcriptional states based on this grouping revealed that nearly all neuronal groups possessed distinct expression motifs (Figure 5B).

**Figure 6:**
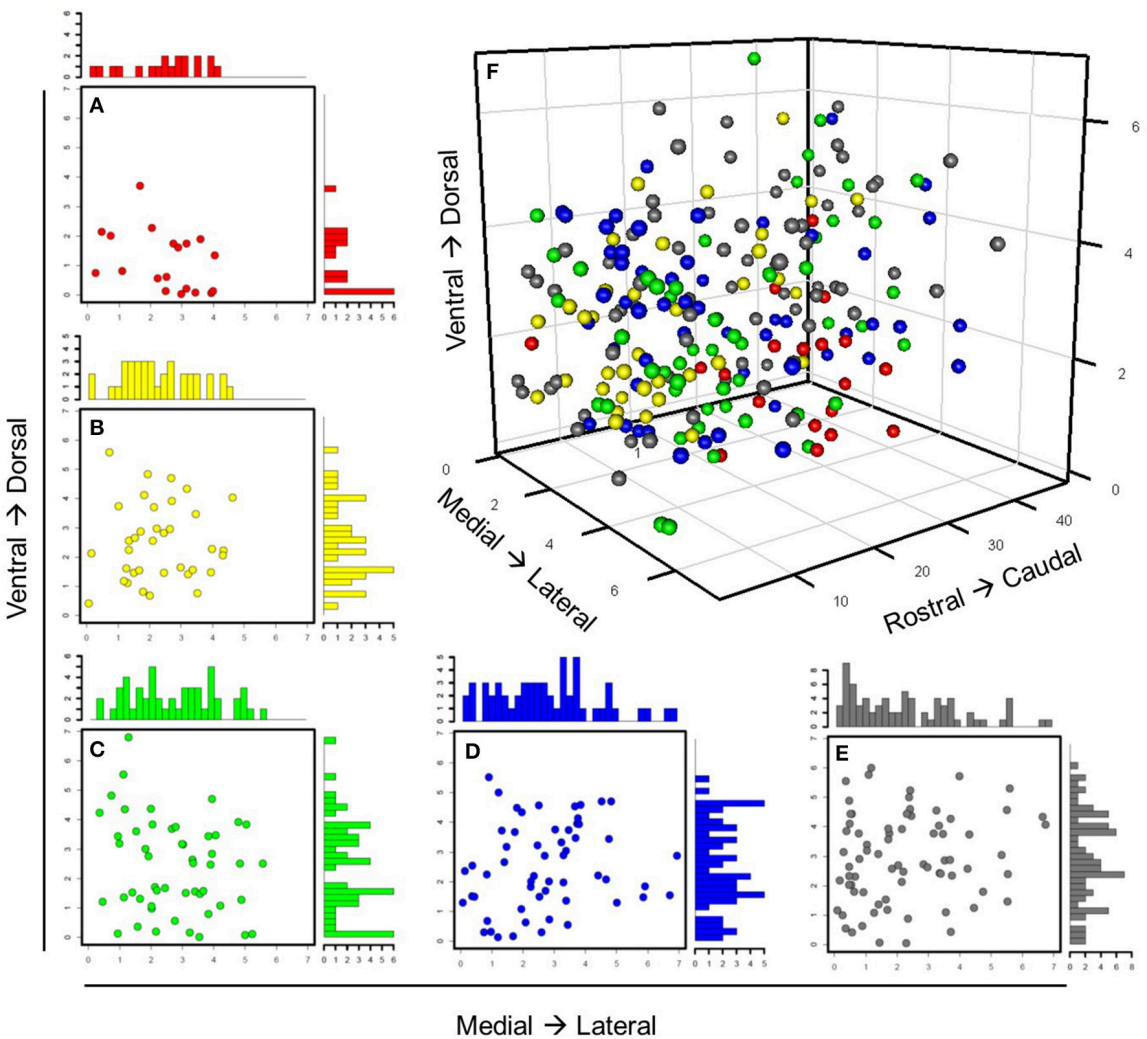
**Spatial distribution of neuronal groups throughout SCN**. Individual neurons are color-labeled with the same color annotation defined in Figure 5. The same anatomical coordinates were used to track spatial positioning of light-treated neurons throughout the SCNs from which they were collected. **(A–E)** Scatterplots with marginal histograms show the locations and relative density of these neuronal groups along the ventrodorsal and mediolateral axes. **(F)** All 235 neurons were plotted with respect to their anatomical position.

In order to verify these neuronal groups, we evaluated how well the expression behavior of these groups aligned with prior knowledge of the intrinsic molecular behavior and regional specificity of SCN neuron-types. Our analysis revealed that gene expression behavior within these groups not only aligned with current understanding of the SCN, but also reflected nuanced expression behavior. Group 1 consisted of 19 neurons that showed high *Vip, Per1, and Per2* expression, suggesting that these neurons responded directly to photic inputs from the RHT (Albrecht et al., 1997; Abrahamson and Moore, 2001; Yan and Silver, 2002; Albrecht, 2012). Concomitantly, immediate early genes including *Fos, Jun, Junb*, and *Egr2* were also upregulated. Group 1 neurons were also predominantly located in the SCN core (Figure 6). Further, upregulated expression of *Vip* and immediate early genes along with the localization of these neurons within the core (Figures 6A,B) agree with prior results that map VIP+ neurons in the SCN core (Antle and Silver, 2005; Ko et al., 2010).

Group 2 included neurons characterized by upregulated expression of *Avp* and *Per2*. Core clock genes were also upregulated including *Cry1, Rora, Rorb, Clock*, and *Arntl1–* all transcriptional regulators for *Avp* and *Per*2, which is co-expressed in AVP+ neurons upon light-induction (Dardente et al., 2002). Upregulated expression of VIP receptor gene, *Vipr2*, in these *Avp*+ neurons further relates our results to those of others showing interactions between VIP+ and AVP+ neurons (Romijn et al., 1997; Abrahamson and Moore, 2001). Alignment between the core clock genes' expression behavior across the LP neuron samples and the previously identified molecular behavior of the SCN lend additional validity to our approach and results. Group 2 neurons tended to localize medially, spanning the ventrodorsal region of the SCN. Although several of these neurons were located more ventrally than expected, given their upregulated expression of *Avp*, Group 2 neurons were mainly located dorsally to Group 1 (Figure S12), which agrees with the known region-specific arrangement of VIP+ and AVP+ neurons in the SCN (Moore and Silver, 1998; Leak et al., 1999).

Group 3 consisted of neurons exhibiting upregulated expression of *Adcyap1*. Since PACAP is produced in the RHT, the unexpected endogenous expression of *Adcyap1* prompted us to investigate the expression behavior of other circadian genes in this group more thoroughly. Cell surface receptor genes including *Avpr1b, Avpr2, Npy1r, and Npy2r* were consistently upregulated across these neurons, suggesting that these neurons are receptive to both AVP and neuropeptide Y (NPY). Analysis of the spatial organization of these neurons revealed that they were distributed throughout the SCN (Figure 6D). This broad spatial distribution was further reflected in their nuanced gene expression behavior, which included gene expression specific to both core and shell regions. Genes traditionally understood to be expressed in the core, such as *Egr2, Cebpb, Jun, Rrad*, and the calbindin-related gene, *Calb2*, were all upregulated. However, other genes associated with core-specific expression such as *Crebbp* and *Creb1*, which are involved in CREB-mediated intracellular signaling (Antle and Silver, 2005; Zhu et al., 2012), were downregulated across a majority of these neurons.

Group 4 neurons were characterized by upregulated expression of *Gabra1 and* the PACAP receptor gene *Adcyap1r1*. Similarly, intracellular signaling genes including *Mapk3, Camk2b*, and *Prkaca* (Zhu et al., 2012) and the neuropeptide signaling gene *Pcsk1n*, which codes for the precursor molecule of the peptide little-SAAS, were upregulated as well. Since little-SAAS has been reported to be involved in intercellular coordination within the SCN (Atkins et al., 2010), this upregulated behavior suggests that Group 4 plays a synchronizing role in the SCN. Similar to Group 3, these neurons did not show any clear spatial organization (Figure 6B).

Finally, the fifth group consisted of 73 neurons not considered transcriptionally similar to those of other groups. However, these neurons did share some similar expression and spatial organizational characteristics associated with Groups 1–4. A subset of Group 5 neurons, for example, expressed levels of *Avp* similar to Group 2 neurons, a majority of which were located dorsomedially within the SCN. Similarly, several Group 5 neurons that exhibited upregulated expression of *Vip* and other core clock genes (e.g., *Per2, Cry1*, and *Clock*) mirrored both expression behavior and ventral localization of Group 1 neurons. However, differences in *Slc12a7* and *Grin2m* expression distinguished these neurons from those of Groups 1 and 2. Likewise, another subset of Group 5 neurons expressed increased levels of *Adcyap1r1* and decreased levels of *Vip* and *Avp*, similar to Group 4 neurons. However, decreased expression of *Pcks1n* differentiated these sets of neurons. It is possible that Group 5 neurons may represent functional variances of neurons within Groups 1–4 and add functional robustness (Paszek et al., 2010) to the coordinated SCN response to photic inputs.

Of the five groups, Groups 1–4 included distinct transcription modules that were associated with upregulated expression of a key neuropeptide gene (Group 1—*Vip*; Group 2—*Avp*; Group 3—*Adcyap1*; and Group 4—*Pcsk1n*), which would appear to support current neurochemical criteria used to describe SCN neurons. However, the current criteria does not fully account for the underlying transcriptional states of these neurons, as evidenced by the presence of Group 5 neurons that express multiple neuropeptides and exhibit nuanced gene expression behavior. Moreover, a correlational analysis of the genes within each transcription module within Groups 1–4 revealed that of the five genes having the highest number of correlations (Pearson correlation coefficient ≥0.5), none were neuropeptide genes (Figure 5A). Furthermore, a quantitative reassessment of the reliability of this multi-genic approach to classify neuronal phenotypes showed that the silhouette scores for the newly annotated groupings of LP neurons in the MDS plane (Figure 5C) did improve (negative to positive scores), indicating better consistency among the transcriptional states within these groups (Figure 5 legend).

In summary, topological analysis of the neuronal-correlation network revealed distinct transcriptomic phenotypes that are likely involved in the phase-shift response in LP neurons. Although the correlational analysis elucidated presumptive functional groups within neuron–interaction networks, this analysis does not provide any insight into possible inter-neuronal signaling mechanisms through which SCN cell networks are maintained. Given the established role that paracrine signaling plays in regulating circadian cycling (Maywood et al., 2011) and the broad spatial distribution of neurons within Groups 3–5 throughout the SCN (Figure 6), which suggests the presence of possible paracrine signaling mediated interactions, we investigated what plausible signaling interactions may be connecting these functional groups.

### Statistical inference of plausible group interactions in neuronal network

**Figure 7:**
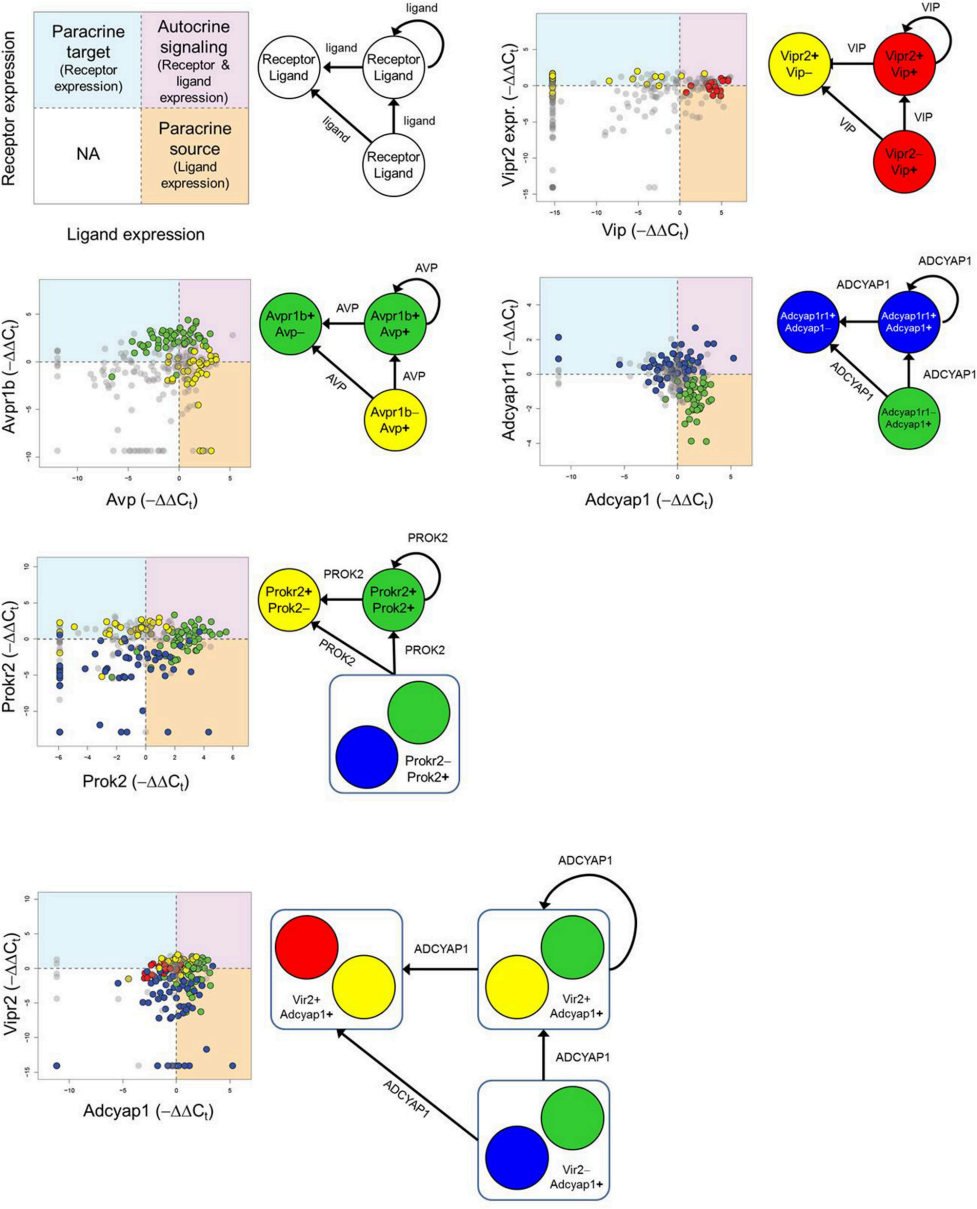
**Inferring neuronal group interactions**. Neurons expressing mRNA for major neuropeptides and their corresponding receptors are identified by −ΔΔC_t_ values and plotted in a bivariate plot. The combinatorial expression of the neuropeptide and receptor genes defines paracrine source/target and autocrine signaling roles. Each quadrant of the bivariate plot in the legend represents a particular signaling role a neuronal group may fulfill. If neuronal group(s) were determined to be statistically enriched within a particular quadrant (Fisher's exact test—Table 1) then the group(s) were deemed to fulfill one or multiple signaling roles: (1) paracrine source (−ΔΔC_t, **ligand**_ > 0 and −ΔΔC_t, **receptor**_ ≤ 0), (2) paracrine target (−ΔΔC_t, **ligand**_ ≤ 0 and −ΔΔC_t, **receptor**_ > 0), or (3) autocrine signaling (−ΔΔC_t, **ligand**_ > 0 and −ΔΔC_t, **receptor**_ > 0) role. A representative set of neuronal group interactions are shown based on the following neuropeptide-receptor pairings: *Avp-Avpr1a, Vip-Vipr2, Adcyap1-Adcyap1r1, Prok2-Prokr2, Adcyap1-Vipr2*.

As part of the functional gene panel used to assess transcriptional states, several neuropeptides and corresponding receptor genes, such as *Vip*–*Vipr2* and *Avp*–*Avpr1b*, were measured. Utilizing this information, we examined the distributions of neuropeptide and corresponding receptor gene expression across the five groups to infer plausible neuronal group interactions. The bivariate expression behavior of neuropeptide–receptor pairs across LP neurons was divided into three distinct expression regimes: *i)* paracrine source (high peptide, low receptor [−ΔΔC_t-peptide_ > 0, −ΔΔC_t-receptor_ ≤ 0]), *ii)* paracrine target (low peptide, high receptor [−ΔΔC_t-peptide_ ≤ 0, −ΔΔC_t-receptor_ > 0]), and *iii*) autocrine signaling (high peptide, high receptor [−ΔΔC_t-receptor_ > 0, −ΔΔC_t-receptor_ > 0]). The regime where low peptide and receptor expression occurred were not considered to play a dominant signaling role within this defined signaling scheme and were therefore ignored (Figure 7). In the interest of focusing on statistically significant group interactions, we used Fisher's exact test to identify which groups, if any, were statistically enriched in each signaling regime and to determine their potential signaling role (Table 1).

**Table 1:** **Statistical enrichment of neuronal groups in signaling roles**.

**Peptide-Receptor pair**	**Group#**	**Paracrine Source**		**Paracrine Target**		**Autocrine Signaling**	
		**Fraction of group**	***p*-val**	**Fraction of group**	***p*-val**	**Fraction of group**	***p*-val**
Avp-Avpr1a	1	0.16	7.60E-01	**0.53**	**9A1E-04**	0.05	9.95E-01
	2	**0.42**	**7.24E-04**	0.08	9.89E-01	**0.39**	**2.13E-02**
	3	0.04	9.99E-01	**0.41**	**5.70E-05**	**0.41**	**1.39E-03**
	4	0.05	9.99E-01	0.09	9.97E-01	0.02I	9.99E-01
	5	**0.32**	**2.20E-03**	0.11	9.95E-01	0.26	3.54E-01
Avp-Avpr1b	1	0.08	8.14E-01	0.19	1.35E-01	0.03	9.93E-01
	2	**0.53**	**4.53E-06**	0.06	9.99E-01	0.28	2.47E-01
	3	0.00	1.00E+00	**0.51**	**1.22E-06**	**0.45**	**2.41E-05**
	4	0.04	9.99E-01	0.21	7.43E-01	0.04	9.99E-01
	5	**0.36**	**4.06E-04**	0.12	9.99E-01	0.22	5.83E-01
Avp-Avpr2	1	0.11	9.19E-01	**0.42**	**3.44E-02**	0.11	9.66E-01
	2	**0.44**	**1.68E-04**	0.06	9.99E-01	**0.36**	**5.13E-02**
	3	0	1.00E+00	**0.49**	**9.52E-07**	**0.45**	**1.15E-04**
	4	0.05	9.99E-01	0.14	9.68E-01	0.02	1.00E+00
	5	0.34	2.10E-04	0.12	9.97E-01	0.23	6.13E-01
Adcyap1-Vipr2	1	0.11	9.81E-01	**0.42**	**1.04E-02**	0.16	9.32E-01
	2	0.08	9.99E-01	**0.44**	**6.07E-05**	**0.42**	**3.10E-02**
	3	**0.49**	**6.69E-05**	0.00	1.00E+00	**0.49**	**1.34E-04**
	4	**0.41**	**4.40E-03**	0.02	1.00E+00	0.02	1.00E+00
	5	0.12	9.99E-01	0.25	6.74E-02	0.27	5.44E-01
Adcyap1-Adcyap1r1	1	0.05	9.99E-01	0.42	8.40E-02	0.21	6.07E-01
	2	0.31	6.66E-01	0.22	7.74E-01	0.19	6.88E-01
	3	**0.94**	**2.15E-26**	0.00	1.00E+00	0.04	9.99E-01
	4	0.04	1.00E+00	**0.41**	**3.35E-03**	**0.39**	**2.90E-04**
	5	0.03	9.99E-01	0.32	2.05E-01	0.30	6.34E-01
Prok2-Prokr2	1	0.21	6.07E-01	0.32	2.36E-01	0.37	1.93E-01
	2	0.00	1.00E+00	**0.53**	**1.35E-05**	0.31	3.10E-01
	3	**0.41**	**1.88E-04**	0.02	9.99E-01	**0.51**	**1.09E-05**
		**0.32**	**2.06E-02**	0.04	1.00E+00	0.02	1.00E+00
	5	0.10	9.99E-01	**0.34**	**3.92E-03**	0.22	8.67E-01

*Bold values correspond to statistically significant interactions (p ≤ 0.05)*.

Consistent with behavior of the established input-output pathway in which photic input stimulates VIP-producing neurons, which in turn stimulate AVP-producing neurons to generate synaptic and molecular outputs, we observed an enrichment of Group 1 neurons as a VIP paracrine source that interacts with the paracrine target neurons of Group 2, which co-express the corresponding VIP receptor, *Vipr2*, and *Avp* (Figure 7). Paracrine feedback signaling between Group 2 and Group 1 via the AVP–AVPR2 pairing was supported by the data as well (Figure 7, Table 1). Moreover, increased expression of glutamate receptors *Grm1* and *Grm5* and glutamate receptor subunit *Grin1* across Group1 neurons suggest that these neurons are receptive to light–induced production of glutamate in the RHT.

In addition to recapitulating known neuron interactions mediated by VIP and AVP, our analysis revealed additional group interactions involving Group 3 neurons. Interactions between Group 3 and Group 2 neurons were supported by upregulated expression of the prokineticin 2 (*Prok2*) and PROK2 receptor, *Prokr2*, pairing (Figure 7). The upregulated expression of *Prok2* across neurons of Group 3 suggest that this group fulfills PROK2–mediated roles including coordinating peptidergic output of the SCN (Cheng et al., 2002; Prosser et al., 2007). Concomitantly, Group 3 neurons exhibited upregulated expression of glutamate receptor *Gria4* and downregulated expression of PACAP-sensitive receptors *Adcyap1r1* and *Vipr2*. The diverging expression behavior of these receptor genes, when considered with the fact that these neurons were co-localized in the ventral region (Figure 6) where the RHT innervates the SCN, may indicate that this group is responsive to glutamate-specific signals generated by the RHT, in response to photic inputs.

Multiple interactions involving Group 4 also hinted at the interconnected nature of the neuronal network within the SCN. A plausible interaction between Group 4 and 3, mediated by the PACAP–ADCYAP1R1 pairing (Figure 7), was supported by the increased expression of *Adcyap1* in Group 3 and of *Adcyap1r1* in Group 4. Upregulated expression of *Adcyap1r1* also supports the possibility that Group 4 neurons respond directly to photic inputs via PACAP released from the RHT (Dziema and Obrietan, 2002; Dragich et al., 2010). Additionally, the increased expression of *Pcsk1n* in Group 4 implies that this group may fulfill a synchronizing role. Previous studies have shown that *Pcsk1n* expression localizes in neurons located centrally within the SCN, overlapping with neurons producing gastrin-releasing peptide (GRP) (Atkins et al., 2010). Although *Grp* expression data was not included in our final analysis (due to assay contamination), assuming that Group 4 neurons co-express *Grp*, upregulated expression of gastrin-releasing peptide receptor (*Grpr*) in Groups 2 and 3 suggests that additional interactions occur between these groups and Group 4, further supporting its integrating role in the SCN.

**Figure 8:**
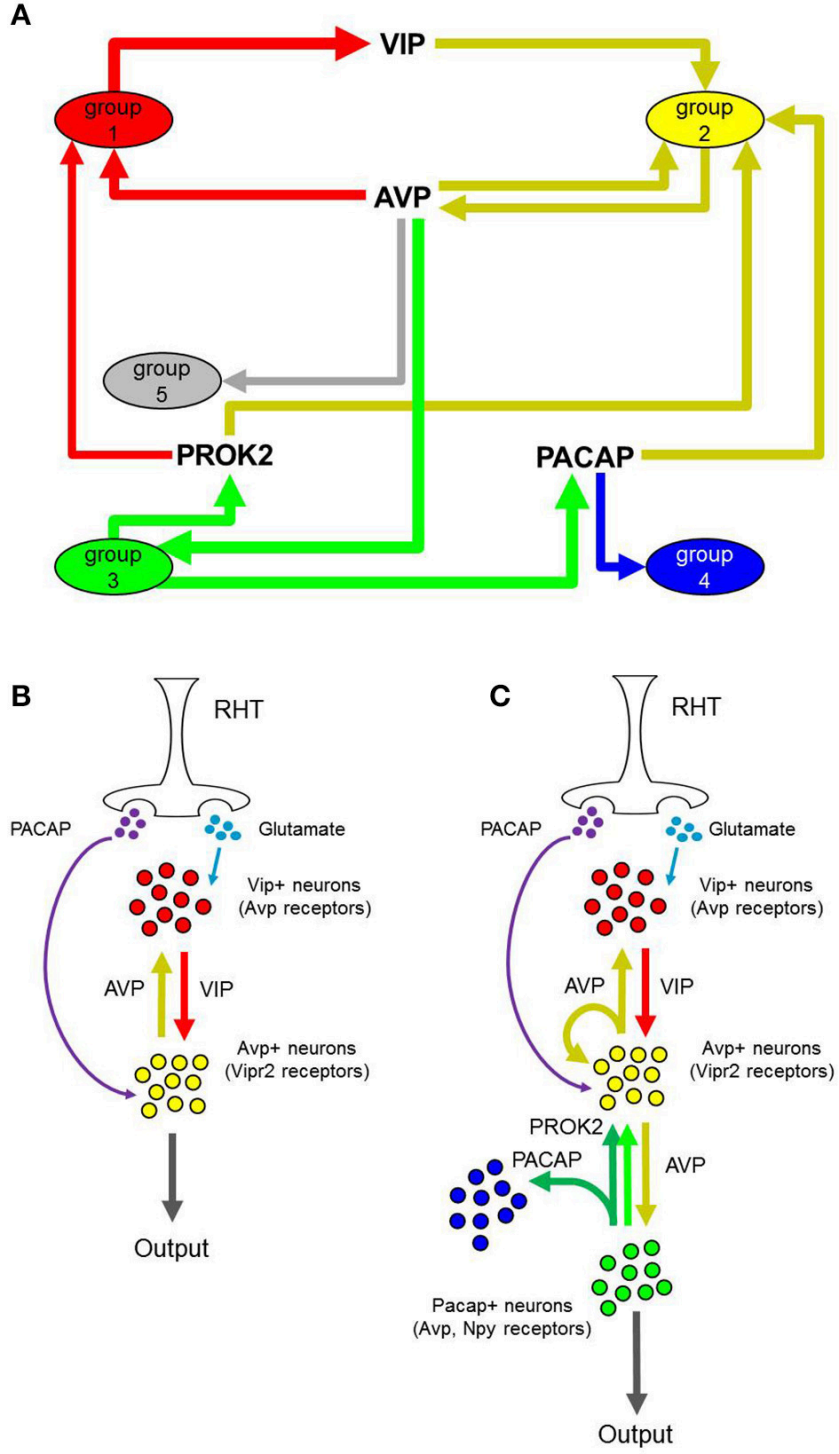
**Proposed inter-neuronal SCN networks and input-output pathways. (A)** Dominant interactions between presumptive neuronal groups. Nodes represent neuronal groups with directional arrows and neuropeptides represent mode of interaction between connected neuronal groups. Edge thickness corresponds to the fraction of single neurons within each group that express a peptide or receptor involved in the interaction. Dominant interactions were identified as those interactions having a Pearson correlation coefficient larger than those determined from random permutations of the peptide-receptor expression data across the LP neurons. **(B,C)** Comparison of one representation of the established input-output pathway of the SCN vs. our proposed input-output pathway, based on the newly identified neuronal groups.

By viewing plausible neuronal interactions in this manner, we can develop a more comprehensive neuron-interaction network that builds upon the established input-oscillator-output system. In addition to recapitulating known interactions between VIP+ and AVP+ neurons (Welsh et al., 2010), our analysis suggests that paracrine signaling mechanisms connect neuronal phenotypes that are independent of the anatomical regions that have, in part, previously defined SCN neuron types (Figure 8).

## Discussion

Characterizing cellular phenotypes is central to understanding how cells function within a network to contribute to and regulate physiological processes, particularly to SCN coordination of circadian rhythms. Although prior work relied on neurochemical criteria and physiological approaches to characterize SCN neuron types, functional heterogeneity across single SCN neurons conflicts with these classification approaches. The work presented here analyzes the transcriptional and spatial organization of single SCN neurons from DD and LP treated mice. By integrating this precise spatial and gene expression data from hundreds of SCN neurons with multivariate mathematical techniques, we identified organizational frameworks (or lack thereof in the case of the DD neurons) that begin to reconcile the heterogeneous behavior observed in SCN neurons in dark-adapted and light-pulsed conditions. The absence of any clear anatomical organization in the DD neurons, relative to the structured anatomical organization between group 1 (*Vip*+) and group 2 (*Avp*+) LP neurons, suggests that the photic input forces a constrained transcriptional response. A diverse array of inputs, including non-hypothalamic neuronal innervations and paracrine signaling neuropeptides (Maywood et al., 2011) are continually influencing and driving neuronal state and function (Park et al., 2014a). The diversity of inputs is reflected in the wide range of transcriptional states exhibited by the DD neurons and random anatomical distribution through the SCN. Because the DD neurons are not receiving photic input, they are not forced to respond to this external constraining force and hence likely exist in a wider range of transcriptional states. Conversely, LP neurons exhibit more constrained and organized transcriptional states, due in part to the photic input received by these neurons. The anatomic organization between Group 1 and 2 supports a spatially biased transcriptional response of SCN neurons to photic input. It is plausible that this spatial organization is due to RHT innervation of the SCN core. Such a cellular input-driven transcriptional response aligns with our previous findings from an analysis of single NTS neurons responding to inputs from baroreceptors sensing changes in blood pressure (Park et al., 2014a).

In addition, we established a classification of SCN neurons, which provided a transcriptional basis from which we could infer cell-interaction network models from a population of heterogeneous neurons contributing to SCN function and robustness (Liu et al., 2007; Ko et al., 2010; Mohawk et al., 2012). Our analysis not only identified known neuronal phenotypes (Group 1–*Vip*+ neurons and Group 2–*Avp*+ neurons), but also revealed additional phenotypes that have not been described previously. While there is an extensive amount of literature detailing the various excitatory and inhibitory signaling mechanisms affecting the SCN, we have focused our single-cell transcriptomic analysis on the neuropeptides and paracrine signaling mechanisms deemed to regulate the circadian clock. Within the context of this paracrine signaling network (Figure 8B), the neuronal phenotypes that arise from our unique single-cell transcriptomic data set represent neuron-types that add complexity to the paracrine signaling network underlying synchronization or circadian clock across the SCN. Our analysis and development of a neuronal network structure provides a model with which to interpret and understand more comprehensively the single-neuron transcriptional heterogeneity pervasive within the SCN.

The multi-genic analysis of SCN neurons revealed greater molecular complexity across a population of SCN neurons than heretofore described (Moore and Silver, 1998; Abrahamson and Moore, 2001; Zhu et al., 2012). This complexity manifested in several ways including *i)* the spatial organization of a subset of *Avp*+ Group 2 neurons, *ii)* the co-expression of *Vip* and *Avp*, and *iii)* the endogenous expression of *Adcyap1* in neurons located throughout the SCN. One possible explanation for the larger than expected spatial distribution of *Avp*+ Group 2 neurons may be due to differences in the rhythmicity between mRNA and peptide expression (Kume et al., 1999). AVP mRNA expression is robustly rhythmic while the corresponding peptide expression is less so. Therefore, some IHC studies have used the drug colchicine to inhibit release and promote accumulation of neuropeptides in the perikarya to enhance staining. Because single-cell qRT-PCR approaches measure mRNA, obviating the need for colchicine, it is possible that this approach revealed a subset of AVP mRNA-expressing neurons that lies outside of the dominant dorsomedial shell region identified by protein staining. Previous studies using *in situ* hybridization to map mRNA expression in the SCN have shown similar localization of AVP mRNA (Card et al., 1988, Figure 6B). While co-expression of *Vip* and *Avp* is not characteristic of SCN neurons, our results and those of others (Romijn et al., 1999; Mieda et al., 2015) do support the idea that *Vip* and *Avp* co-expression does occur within a small subset of SCN neurons. Finally, the precise nature of LCM sampling (Park et al., 2014a) and the large spatial distribution of *Adcyap1*+ neurons throughout the SCN suggest that these results are not an artifact of any unlikely residual RHT processes contaminating the samples.

Despite the central roles neuropeptides play in circadian regulation, our gene correlational network analysis suggested that neuronal states are driven more by the inputs they receive rather than the peptidergic outputs they produce. We have observed similar input-driven functional organization of neuronal states in other brain nuclei (Park et al., 2014a) as well. Concurrently, this receptor-based transcriptional regulation occurring in SCN neurons may reflect a neuron's ability to sensitize itself to specific inputs. This sensitization or “gating” modulates SCN responsiveness to photic inputs at specific times during the circadian cycle (Gillette and Mitchell, 2002; Abbott et al., 2013; Iyer et al., 2014). Because transcriptional profiles were measured early in the dark cycle (ZT15–a time when SCN neurons are more sensitive to phase–shifting photic inputs), it is possible that receptor–correlated expression reflects this gating behavior *in vivo* (Gillette and Mitchell, 2002).

This analysis showed that nearly all neurons were associated with a particular neuronal phenotype, with the exception of Group 5 neurons. While we hypothesize that Group 5 may play multiple roles in the neuron-interaction network, the presence of these neurons brings to light some limitations of our sample set. Given the complex and dynamic nature of circadian regulation, SCN neurons are continually responding to multiple input types. Had we measured single-neuron transcriptional profiles at multiple times, our analysis would have likely revealed neuronal groups distinct from those identified herein. Moreover, while the underlying transcriptional organization of most of the groups identified was independent of animal-animal variability, Group 3 was composed predominantly of neurons taken from one animal subject (Figure S13). Given the extent of transcriptional heterogeneity observed, which in many cases surpass animal-animal variability (Durruthy-Durruthy et al., 2014; Park et al., 2014a; Llorens-Bobadilla et al., 2015), it is surprising to see such a concentration of neurons from one animal as no experimental biases or particular behavioral or physiological phenotypes were identified. Further, single-cell RNA sequencing of SCN neurons would have provided a comprehensive perspective of the transcriptional states of SCN neurons and potentially lead to the identification of additional neuron-types not included in our analysis.

However, the main intent of this work was to provide a molecular framework from which to interpret the single-cell heterogeneity of SCN neurons. Given that the transcriptional components underlying circadian rhythms are well characterized (Sato et al., 2003; Araki et al., 2006; Doherty and Kay, 2010), our analysis focused on these and other functionally relevant genes to develop an organizational framework sufficient enough to reconcile SCN function and single-neuron transcriptional heterogeneity. While the data used in this study may not have provided a completely comprehensive analysis of the transcriptional states of SCN neurons, this study, along with previous efforts (Park et al., 2014a; Darmanis et al., 2015), suggests that the depth and breadth of the gene panel manually curated here was able to infer such an organizational framework. Given these limitations, further investigation—including single-cell RNAseq analysis across multiple time points in which photic input sensitivity and light/dark cycle durations differ—would provide valuable information regarding the function and organization of SCN networks.

The ability to interpret how single cells organize into functional interaction networks from heterogeneous cellular behavior not only provides fundamental insight into SCN organization, but may also provide similar insights into the molecular organization of cellular networks and neuronal circuits of other tissues that regulate stable steady states (i.e., homeostasis). These neuronal circuits undergo distributed rearrangements throughout life yet are able to maintain stable behavior within an environment of continual perturbations (Maffei and Fontanini, 2009). Previous work has investigated how neuronal circuits and networks configure and self-regulate via synaptic connections, synaptic scaling, and permissive signaling that modulate cellular behavior (Maffei and Fontanini, 2009; Davis, 2013). And while current efforts continue to yield insight into network homeostasis, questions regarding how coordinated changes and phenotypic modulation that regulate network homeostasis remain. “The challenge is to begin assembling an emerging molecular ‘parts list’…” and “[specify] cell identity,” in order to understand how neural activity is regulated (Davis, 2013). Our approach to analyze and develop a data-driven cellular network structure provides a methodology with which to interpret how single-cell heterogeneity contributes to cell network and neuronal circuit formation and has the potential to provide such insight.

## Concluding remarks

Our results show distinct functional phenotypes that exist outside the traditional neurochemical definitions of the SCN. Even though these neurochemical criteria are useful and conveniently describe key aspects of SCN function, they fall short of fully capturing the complexity and diversity of the neuronal components driving SCN function. Although our findings are limited to specific times chosen for this study, our approach provides a unique perspective of SCN functional networks that provide plausible explanations as to how these neuronal phenotypes and neuron-neuron interactions organize under dark-adapted and phase-shifting behavior.

## Data access

Both raw C_t_ and −ΔΔC_t_ values of samples passing quality control are included as supplemental text files (Table S1 and S2).

## Author contributions

JP performed analysis, figure generation, and was a main contributor to the writing of the manuscript. HZ conducted experiments and guided sample collection, performed analysis, and contributed to writing and figure design. SO contributed to analysis and writing. BO was involved in initial analysis and editing. DW, JS, and RV designed the study and were involved in analysis, figure design, and editing. JP and HZ contributed equally to the study. All authors discussed the results and commented on the manuscript.

### Conflict of interest statement

The authors declare that the research was conducted in the absence of any commercial or financial relationships that could be construed as a potential conflict of interest.
