# Supplementary material for: Single-Cell Transcriptional Analysis Reveals Novel Neuronal Phenotypes and Interaction Networks Involved in the Central Circadian Clock
Source: Front Neurosci. 2016 Oct 25;10:481. doi: 10.3389/fnins.2016.00481 (PMC5079116; doi:10.3389/fnins.2016.00481)
Supplement: Supplementary file 1 [file Presentation1.PDF]

TITLE: Deciphering Single-Neuron Phenotypes and Interaction Networks Involved in Circadian Regulation

Supplemental Information

James Park[1,2†], Haisun Zhu[1†], Sean O'Sullivan[1], Babatunde Ogunnaike[2], David R. Weaver[3], James Schwaber[1,2]* and Rajanikanth Vadigepalli[1,2]*

[1]Daniel Baugh Institute for Functional Genomics and Computational Biology, Department of Pathology, Anatomy and Cell Biology, Sidney Kimmel Medical College, Thomas Jefferson University, Philadelphia, Pennsylvania

[2]Department of Chemical and Biochemical Engineering, University of Delaware, Newark, Delaware

[3]Department of Neurobiology, University of Massachusetts Medical School, Worcester, Massachusetts

Contact:

James Park	parkjams@udel.edu
Haisun Zhu	haisun.zhu@gmail.com
Sean O'Sullivan	sean.o'sullivan@jefferson.edu
Babatunde Ogunnaike	ogunnaik@udel.edu
David R. Weaver	david.weaver@umassmed.edu
James Schwaber	james.schwaber@jefferson.edu
Rajanikanth Vadigepalli	rajanikanth.vadigepalli@jefferson.edu

*Correspondence to: james.schwaber@jefferson.edu
rajanikanth.vadigepalli@jefferson.edu

†contributed equally to the present work

Principal Component Analysis (PCA)

The multivariate PCA technique reduces data dimensionality (here 87 genes correspond to 87 dimensions) by determining new coordinates in a transformed lower dimensional space defined by uncorrelated coordinate axes or principal components. The principal components are structured and ordered in such a way that the greatest amount of variation in the original data is captured along the first principal component, the next greatest amount by the second component, and so forth such that the least amount of variation is captured in the last principal component. This dimensionality reduction, or simplification, allows us to describe the data with a few informative component axis, which helps to reveal hidden structures or patterns within the data. In this case, structures or clusters of transcriptional states would be interpreted as distinct cellular phenotypes. We found that the first three principal components retained 46% of the original transcriptional data variability (Fig. 2B-D), which was sufficient to distinguish dark-adapted neurons from those treated with the light pulse at ZT14.

To understand the relative contributions of the measured genes to the variability in the data, we examined the corresponding principal coordinate system, onto which light-treated neurons were projected. Each principal component consisted of weighted contributions from all 87 genes. The correlation of a gene to a given principal component is referred to as a “loading”, which represents the contribution of the gene to the positioning of the transcriptional state along this component. Thus, the larger the magnitude of the loading, the greater the contribution a gene has to the variability along that component.

PCA was performed using MultiExperiment Viewer (MeV; (Howe et al., 2010))

Quality assessment of data

Due to the region-specific expression nature of certain SCN markers, we expected several samples to show minimal to no expression of the corresponding genes (e.g. *Vip, Avp*). Therefore, individual reactions that failed were interpreted to represent either too low or no measurable amount of cDNA in the amplified sample. Consequently, a “minimum-1” value was substituted for that particular qRT-PCR reaction (the minimum value being the lowest -$\Delta\Delta$Ct value across all DD and LP single neuron samples for a particular gene) (Bergkvist et al., 2010).

Gene correlation networks

A Pearson correlation coefficient threshold of 0.5 was used to define a significant relationship between a gene pair. This correlation value of 0.5 was empirically verified as a significance threshold using repeated random permutations of the gene expression data within the various defined cell-types (Fig. 4B-D, Fig. S4-S8). The high number of correlations between receptors and genes versus the low number or absence of correlations between the neuropeptides of interest and genes suggest that gene regulatory behavior in SCN neurons is driven more by the neuropeptide inputs they receive rather than the neuropeptide output they produce. In other words, the expression of key circadian neuropeptide genes like *Vip*, *Avp*, and *Adcyap1* does not appear to be principal drivers of underlying transcriptional states of SCN neurons experiencing a phase-shift.

Multidimensional Scaling (MDS)

Non-metric MDS was performed in order to map the high-dimensional data set of dissimilarity values to a new set of “configuration” points $x_i, \dots, x_n$ in a lower k-dimensional space such that the rank-order of the dissimilarity values between a pair of single cells is well-approximated by the rank-order of distance values in the lower k-dimensional space. MDS is a technique that has been used extensively for classification in gene expression studies (Fuller et al., 2002; Ross et al., 2003; Taguchi and Oono, 2005). Consequently the MDS technique maps these dissimilarity values into a lower dimensional space by interpreting the dissimilarity values as distances, which in this case equates to coordinate distances in a 3-D space. Therefore the Euclidean distance between each pair of spheres (i.e. single cells) represents the difference in their relative gene expression behavior. The closer the cells are in this projected space, the more similar they are in their transcriptional behavior. MDS aids in the visualization of the dissimilarity value between pairs of single cells and the distances between each mapped single cell approximates the calculated pair-wise dissimilarity calculated among the single cell samples. This mapping procedure occurs while minimizing the error between the actual distances in the original n-dimensional space and the lower dimensional space (i.e. minimizing the stress) (Van Deun and Delbeke, 2000). This error, or stress (S), is defined as:

$$S = \sqrt{\frac{\sum_{ij}\left(d_{ij} - d_{ij}^{*}\right)^2}{\sum_{ij} {d_{ij}^{*}}^2}}$$

Where d_{ij} is the target distance in the original n-dimensional space and d_{ij}^{*} is the configuration distance in the lower k-dimensional space.

Configuration points were determined using the *isoMDS* function within the *MASS* package provided by R (R Core Team, 2013).

Supplement Table and Figure Legends

Table S1. Raw C_t data

Table S2. $-\Delta\Delta C_t$ data

Table S3. Gene symbols and Primer Designs

Table S4. Number of pairwise gene Pearson correlations across dark-adapted neurons. Only positive pairwise gene correlations across with coefficient values determined to be of statistical significance (> 0.25, ref Fig. S3) were counted.

Figure S1. Technical replicability. A serial dilution sample set of RNA extracted from a mouse hypothalamus was assayed on the 96.96 BioMarkTM dynamic arrays. A pairwise comparison among all dynamic arrays showed that the C_t values for the dilution sample sets fall along the 45 degree line (black dashed line) with minimal deviation, indicating minimal variability from chip to chip. The slope values obtained from replicability across chips range from 0.93 – 0.96 and R^2 values range from 0.90 – 0.91.

Figure S2. Heat map of dark-adapted neurons. A visualization of the normalized gene expression of all genes (passing quality control assessment) across all dark-adapted neurons.

Figure S3. Empirical determination of significance threshold for Pearson correlation coefficient values in neurons from dark-adapted mice. Normalized gene expression data was permuted over 1000 iterations to determine the distribution of gene-gene correlations across the 88 cells collected from dark-adapted mice Given the sample size and variability in gene expression data present across the dark-adapted neurons, the empirically determined distribution of correlation coefficients suggests that Pearson correlation coefficient values >= 0.25 are statistically significant to a p-value of 0.053, marked by the red dashed line. Therefore positive Pearson correlation coefficients >= 0.25 determined within the dark-adapted neuronal data set are not likely to be a result of random chance. The table below lists the number of significant correlations shared by the tabulated genes, which were depicted in the heat map (Fig. 1D). The table below lists the corresponding p-value, empirically determined, for the various Pearson correlation coefficient values from the permuted data.

Figure S4. Principal component analysis (PCA) of neurons from dark-adapted mice. **(A)** Scores plot along first three principal components obtained from PCA of transcriptional profiles of dark-adapted neurons (dark-grey spheres). The normalized $-\Delta\Delta C_t$ values were used in the PCA. Corresponding loading values of genes along PC 1 v. PC 2 **(B)**, PC 1 v. PC 3 **(C)**, and PC 2 v. PC 3 **(D)** are represented in the 2D plots. The labeled genes have a larger contribution to the variability observed in the dataset. The scattered positioning of the neurons in (A) suggest that several neurons cluster together and that there may be some transcriptional organization to these dark-adapted neurons.

Figure S5. Spatial distribution of *Adcyap1* expressing neurons. The combined scatterplot and histograms show light-treated cells with positive expression of Adcyap1 ($\Delta\Delta C_t > 0$) are not localized to a particular location and are distributed throughout the SCN. The scatterplot depicts the ventrodorsal and mediolateral position of the single cells. Red-outlined circles indicate single neurons that display positive Adcyap1 expression. The corresponding histograms show the distribution of cells with positive Adcyap1 expression along the ventrodorsal and mediolateral axes.

Figure S6. Empirical determination of significance threshold for gene correlation network (light-pulsed cells). Normalized gene expression data was permuted over 1000 iterations to determine the possibility of generating a gene-gene correlation equivalent to or greater than those found in the data (i.e. pairwise gene Pearson correlation coefficient >= 0.5). For each permutation, Pearson correlation coefficients were calculated for all pairs of genes. Histograms represent distributions of these Pearson correlation coefficients between various functional relationships including correlations shared with receptor genes and all the neuropeptide genes measured (*Adcyap1, Avp, Pcsk1n, Prok2,* and *Vip*). The dashed red line represents the correlation coefficient threshold used in generating the gene correlation network (Figure 4). These distributions were determined for gene-gene correlations within subset of SCN neurons including **(A)** *Adcyap1*+ neurons, **(B)** *Adcyap1r1*+ neurons, **(C)** *Avp*+ neurons, **(D)** *Avpr1a*+ neurons, **(E)** *Avpr1b*+ neurons, **(F)** *Avpr2*+ neurons, **(G)** *Vip*+ neurons, and **(H)** *Vipr2*+ neurons. None of the iterations resulted in a Pearson correlation coefficient equal to or larger than the correlations represented in the gene networks (p=0.000).

Figure S7. Gene correlation network within *Adcyap1r1*+ cells and *Avpr1a*+ cells. Gene correlation network across all genes in single SCN neurons. Only correlative relationships (edges) with a Pearson correlation coefficient greater than 0.5 between genes (nodes) are included in network. Nodes are arranged in an identical manner as in Figure 4 and node colors represent median expression values of genes within the respective subsets of light-treated SCN neurons. **(A)** Gene correlation network across genes in single neurons expressing *Adcyap1r1* at levels greater than or equal to the normalized median expression level of *Adcyap1r1* across all cells. Receptor genes such as *Prokr2*, *Vipr2*, *Avpr1a*, *Drd1a,* and *Grin2c* show a greater number of correlative relationships among other genes than neuropeptide genes *Vip, Avp*, and *Adcyap1*. **(B)** Gene correlation network across all genes in single neurons expressing *Avpr1a* at levels greater than or equal to the normalized median expression level of *Avpr1a* across all cells. Receptor genes such as *Npy1r, Npy2r, Npy5r*, *Avpr2*, *Avpr2a, Prok2*, and *Vipr2* show a large number of correlative relationships among other genes suggesting that receptor gene expression plays a strong regulatory role driving the transcriptional states of specific SCN cell-types. Gene correlation networks were constructed using Cytoscape® version 2.8.4.

Figure S8. Gene correlation network within *Avpr1b*+ cells and *Avpr2*+ cells. Gene correlation network across all genes in single SCN neurons. Only correlative relationships (edges) with a Pearson correlation coefficient greater than 0.5 between genes (nodes) are included in network. Nodes are arranged in an identical manner as in Figure 4 and node colors represent median expression values of genes within the respective subsets of light-treated SCN neurons. **(A)** *Avpr1b* at levels greater than or equal to the normalized median expression level of Avpr1b across all cells. Receptor genes such as *Npy2r, Npy5r, Vipr2, Avpr1a,* and *Avpr2a* show a large number of correlative relationships among other genes suggesting that receptor gene expression plays a strong regulatory role driving the transcriptional states of specific SCN cell-types. **(B)** Gene correlation network across all genes in single SCN neurons expressing *Avpr2* at levels greater than or equal to the normalized median expression level of *Avpr2* across all cells. Receptor genes such as *Npy2r, Npy5r, Avpr1b*, and *Avpr2* show a greater number of correlative relationships among other genes than *Vip, Avp,* or *Adcyap1*. Moreover, *Vipr2, Prokr2,* and *Grin2c* also show a large number of correlative relationships further suggesting the regulatory relationships underlying specific cell-types are driven by the inputs the cell-type is responding to rather than the ligand or signal it is generating. Gene correlation networks were constructed using Cytoscape® version 2.8.4.

Figure S9. Gene correlation network within *Vipr2*+ cells. Gene correlation network across all genes in single SCN neurons expressing *Vipr2* at levels greater than or equal to the normalized median expression level of *Vipr2* across all cells. Only correlative relationships (edges) with a Pearson correlation coefficient greater than 0.5 between genes (nodes) are included in network. Node colors represent median expression value of the gene across *Vipr2*+ light-treated cells. Nodes are arranged in an identical manner as in Figure 4. Receptor genes such as *Npy2r, Npy5r, Avpr1b*, and *Avpr2* show a greater number of correlative relationships among other genes *than Vip, Avp,* and *Adcyap1*. Gene correlation networks were constructed using Cytoscape® version 2.8.4.

Figure S10. Neuronal hierarchical clustering (light-pulsed neurons) (A) Dendrogram of light-treated cells based on the Pearson correlation coefficient distance. The cell-type definition based on neuropeptide gene expression does not align with hierarchical clustering indicating that the neuropeptide-based classification does not fully characterize

transcriptional states of single neurons. Additional qualitative assessments of the ability of the Vip/Avp/Adcyap1 expression-based categorization to cluster transcriptional states included the use of a minimum spanning tree **(B)** and a 2-dimenionsal MDS **(C)**. The colors in the MDS plot correspond to the cluster colors in Figure 3A and 3C. Silhouette scores for each cluster represent an average of the silhouette scores calculated for each member within an assigned cluster (Materials and Methods) – cluster 1 (-0.509), cluster 2 (-0.085), cluster 3 (-0.181), cluster 4 (-0.049), cluster 5 (-0.175), cluster 6 (-0.047), cluster 7 (0.104), cluster 8 (-0.075).

Figure S11. Empirical determination of significance threshold for neuronal correlation network. Normalized gene expression data was permuted over 1000 iterations to determine the possibility of generating a neuron-neuron correlation equivalent to or greater than those found in the data (i.e. pairwise neuron Pearson correlation coefficient >= 0.5). For each permutation, Pearson correlation coefficients were calculated for all pairs of light pulsed (LP) neurons. The two histograms represent distributions of these Pearson correlation coefficients between various functional relationships determined from permuting gene expression across genes within a single neuron sample. None of the iterations resulted in a Pearson correlation coefficient equal to or larger than the correlations represented in the neuronal correlation network (p=0.000).

Figure S12. Spatial distribution of Vip+ Group 1 neurons and Avp+ Group 2 neurons. Neurons (spheres) are plotted according to their spatial coordinates within the SCN recorded during sample collection (Material and Methods). Group 1 neurons (red) are predominantly ventral to the Group 2 neurons (yellow), aligning with the known organization of VIP+ and AVP+ neurons in the SCN.

Figure S13. Animal variability across neuron correlation network. The same neuron-neuron correlation network (Figure 5A) is annotated with the animal source from which the single neurons were sampled. Each group is outlined with their corresponding group color as in Figure 5A. Although most groups are composed of neurons from multiple light-treated mice, Group 3 neurons were composed predominantly of neurons from Animal 2.

Supplemental References

Bergkvist, A., Rusnakova, V., Sindelka, R., Garda, J. M. A., Sjögreen, B., Lindh, D., et al. (2010). Gene expression profiling--Clusters of possibilities. *Methods* 50, 323–35. doi:10.1016/j.ymeth.2010.01.009.

Van Deun, K., and Delbeke, L. (2000). Multidimensional Scaling. Available at: http://www.mathpsyc.uni-bonn.de/doc/delbeke/delbeke.htm.

Fuller, G. N., Hess, K. R., Rhee, C. H., Yung, W. K. A., Sawaya, R. a, Bruner, J. M., et al. (2002). Molecular classification of human diffuse gliomas by multidimensional scaling analysis of gene expression profiles parallels morphology-based classification, correlates with survival, and reveals clinically-relevant novel glioma subsets. *Brain Pathol.* 12, 108–16. Available at: http://www.ncbi.nlm.nih.gov/pubmed/11771519.

Howe, E., Holton, K., Nair, S., Schlauch, D., Sinha, R., and Quackenbush, J. (2010). “MeV: MultiExperiment Viewer,” in *Biomedical Informatics for Cancer Research*, 267–277. doi:10.1007/978-1-4419-5714-6.

R Core Team (2013). R: A Language and Environment for Statistical Computing. Available at: http://www.r-project.org/.

Ross, M. E., Zhou, X., Song, G., Shurtleff, S. a, Girtman, K., Williams, W. K., et al. (2003). Classification of pediatric acute lymphoblastic leukemia by gene expression profiling. *Blood* 102, 2951–9. doi:10.1182/blood-2003-01-0338.

Taguchi, Y.-H., and Oono, Y. (2005). Relational patterns of gene expression via non-metric multidimensional scaling analysis. *Bioinformatics* 21, 730–40. doi:10.1093/bioinformatics/bti067.
