# Supplementary figures and images for: Single-Cell Transcriptional Analysis Reveals Novel Neuronal Phenotypes and Interaction Networks Involved in the Central Circadian Clock
Source: Front Neurosci. 2016 Oct 25;10:481. doi: 10.3389/fnins.2016.00481 (PMC5079116; doi:10.3389/fnins.2016.00481)

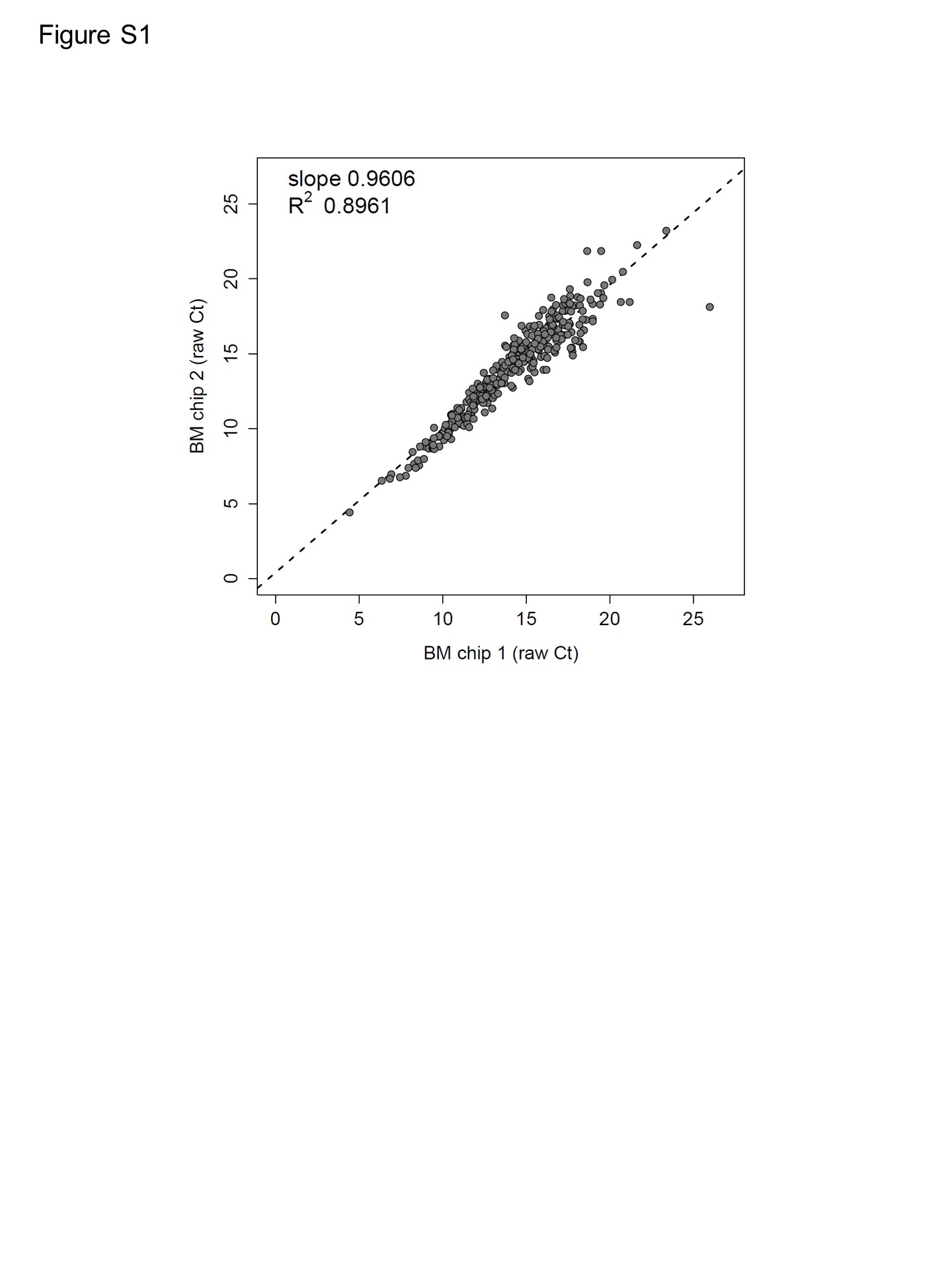

Supplement: Supplementary file 6 [file Image1.JPEG]

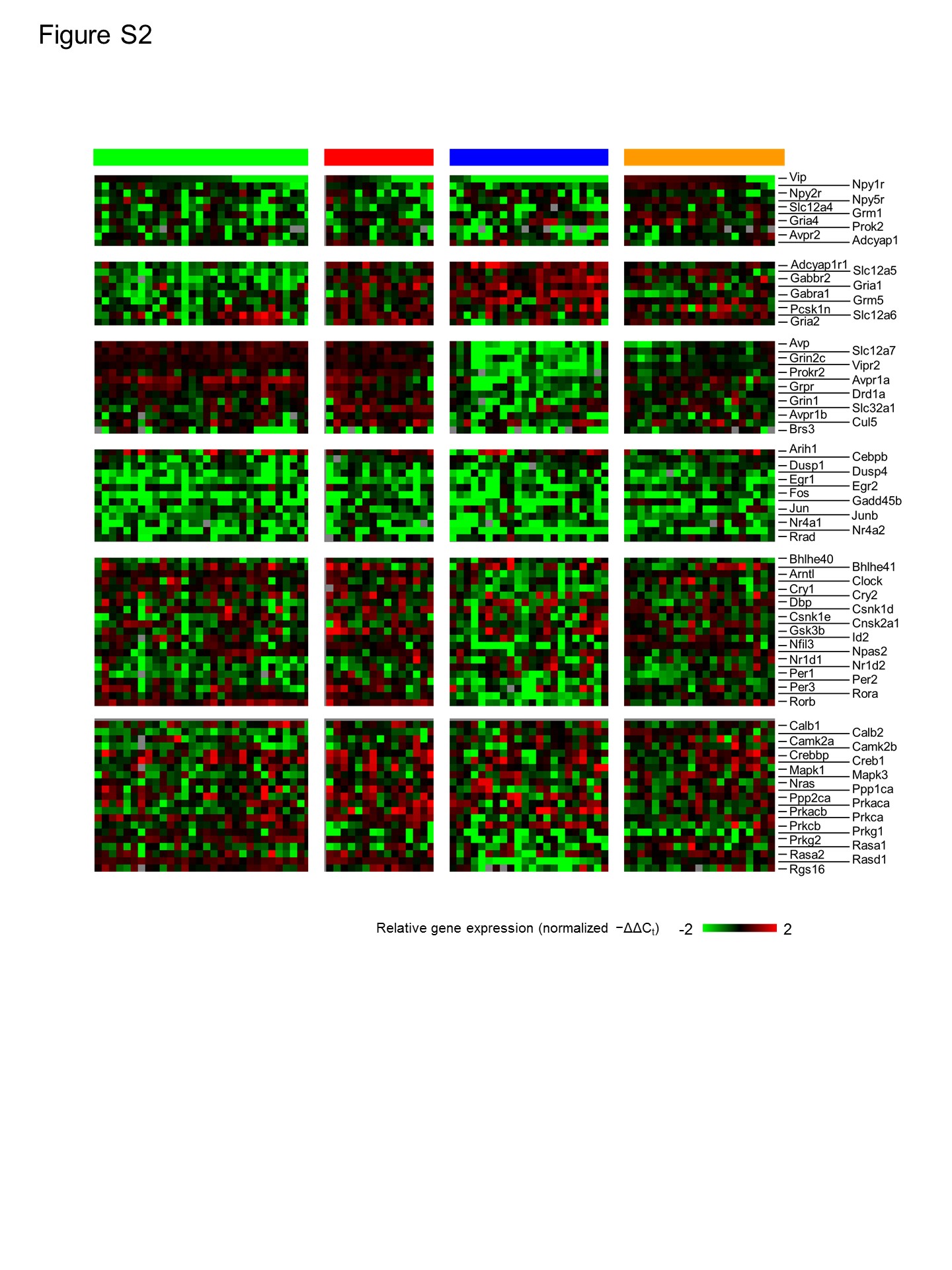

Supplement: Supplementary file 7 [file Image2.JPEG]

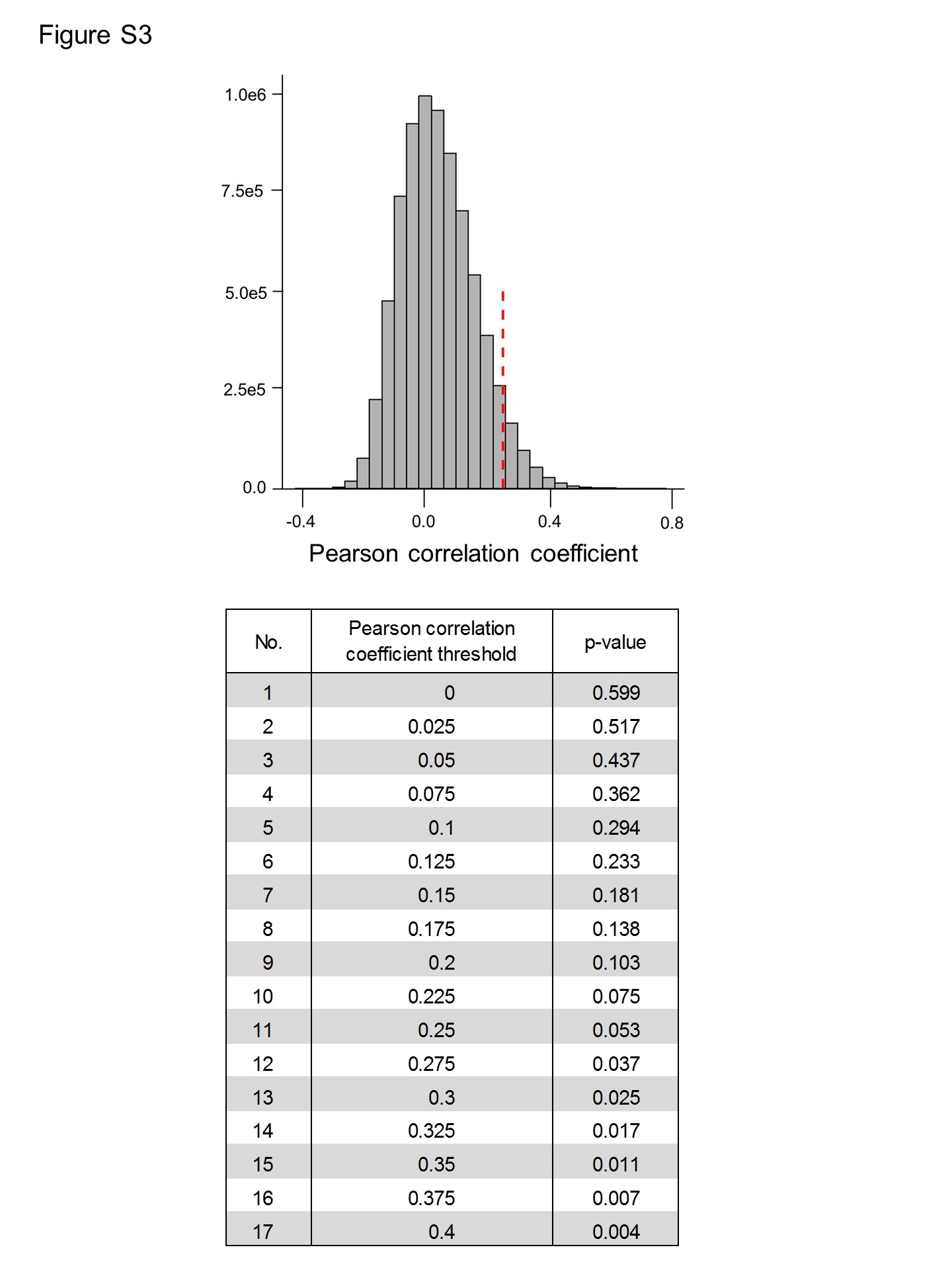

Supplement: Supplementary file 8 [file Image3.JPEG]

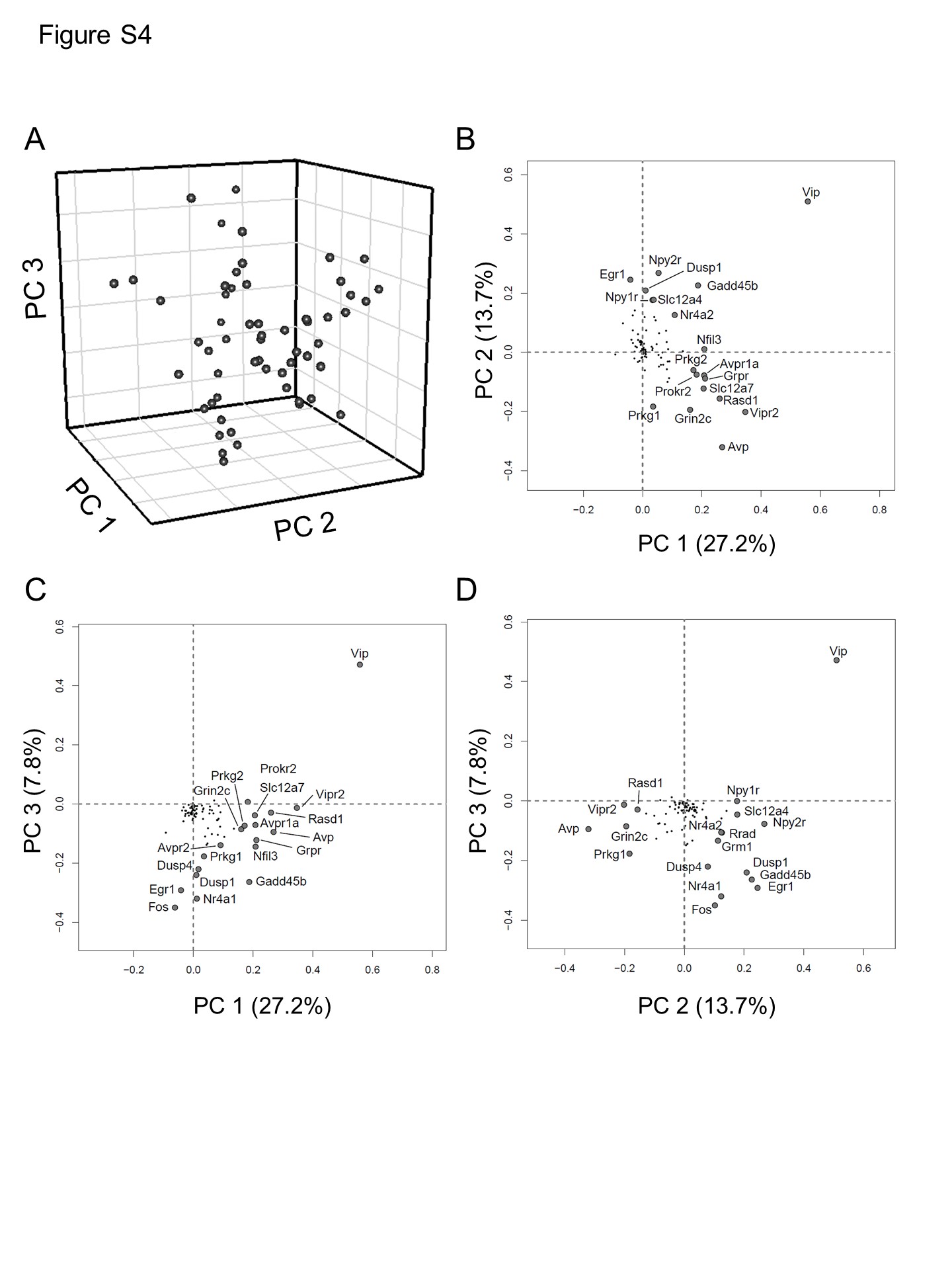

Supplement: Supplementary file 9 [file Image4.JPEG]

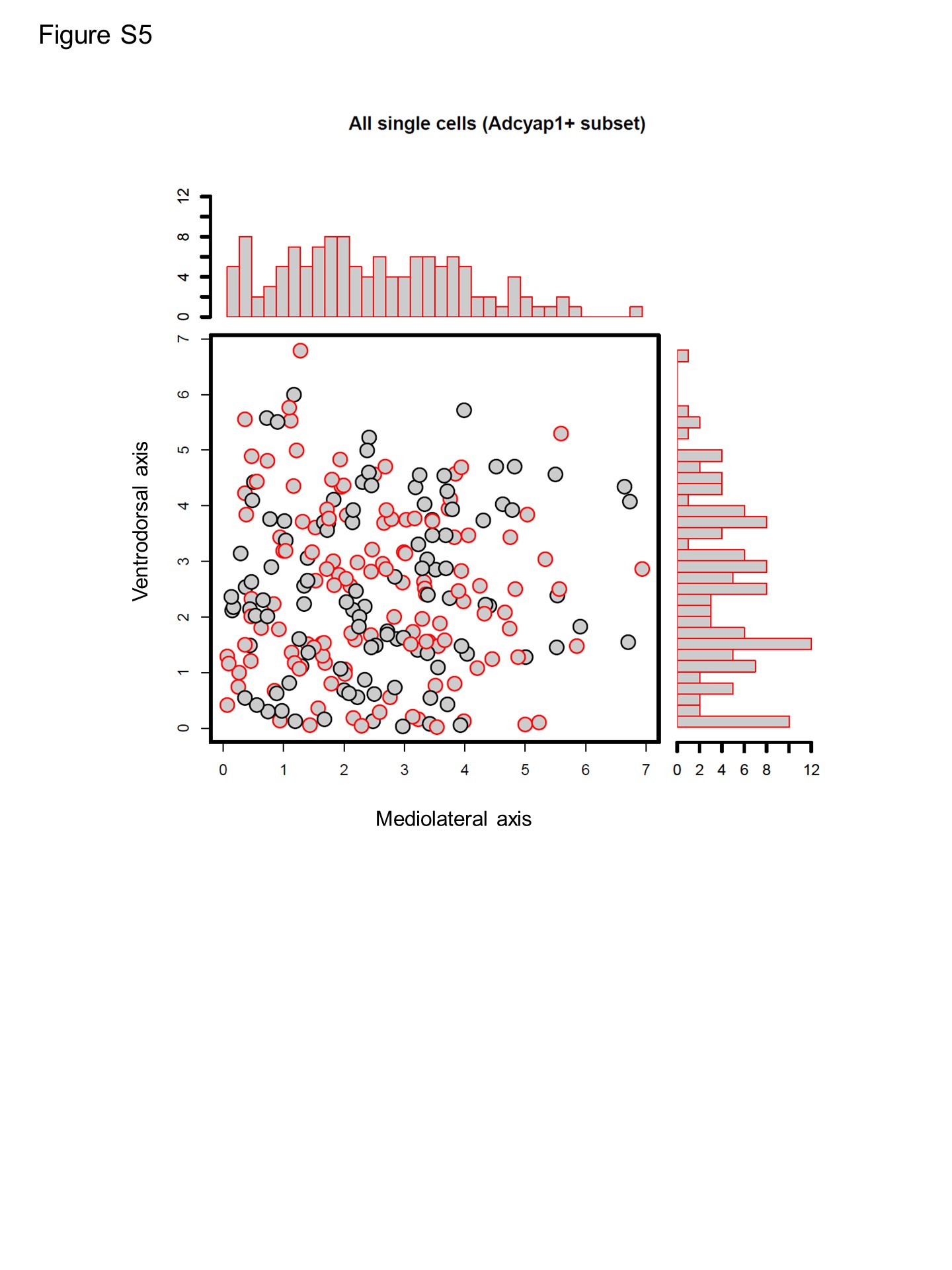

Supplement: Supplementary file 10 [file Image5.JPEG]

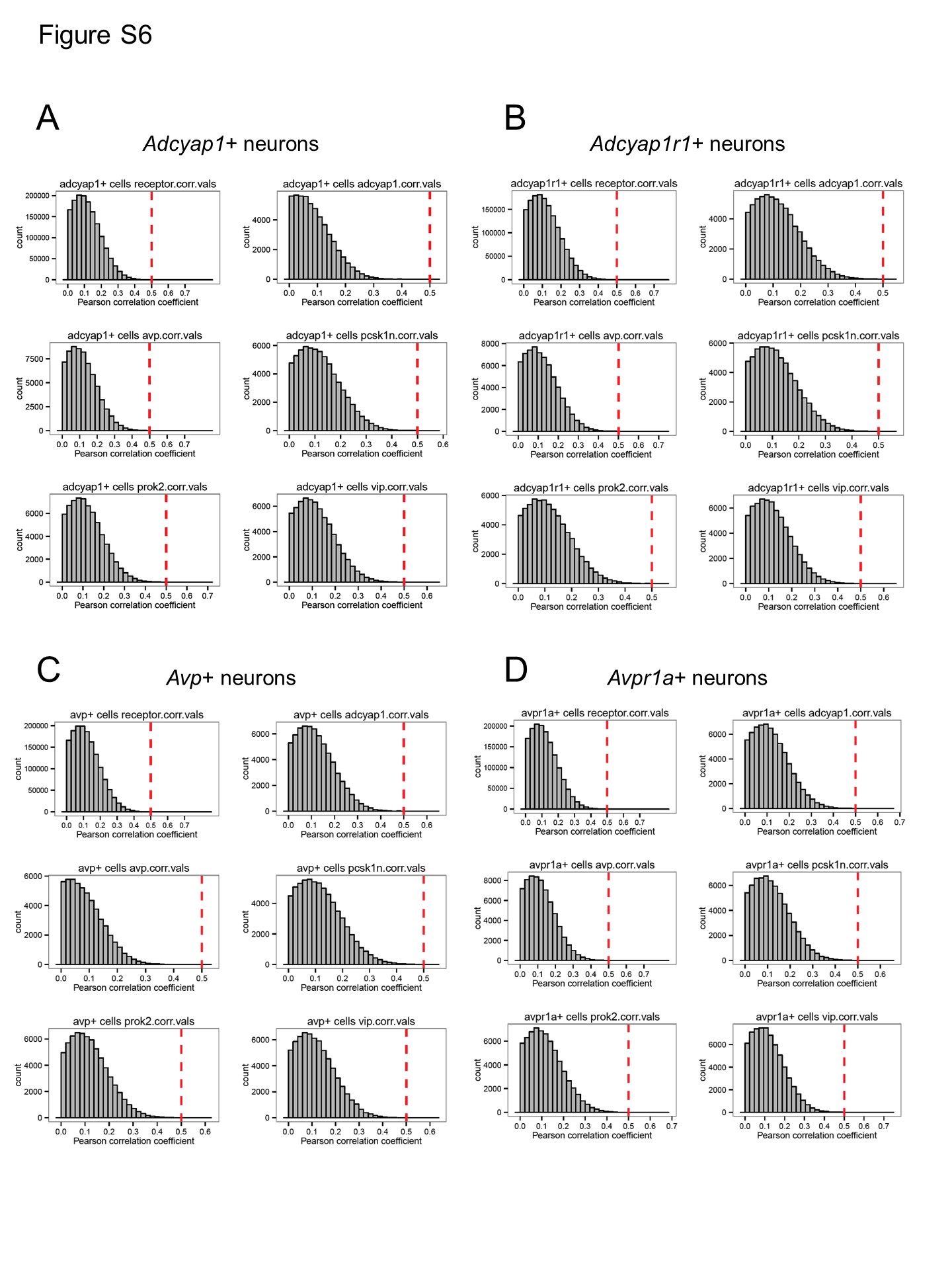

Supplement: Supplementary file 11 [file Image6.JPEG]

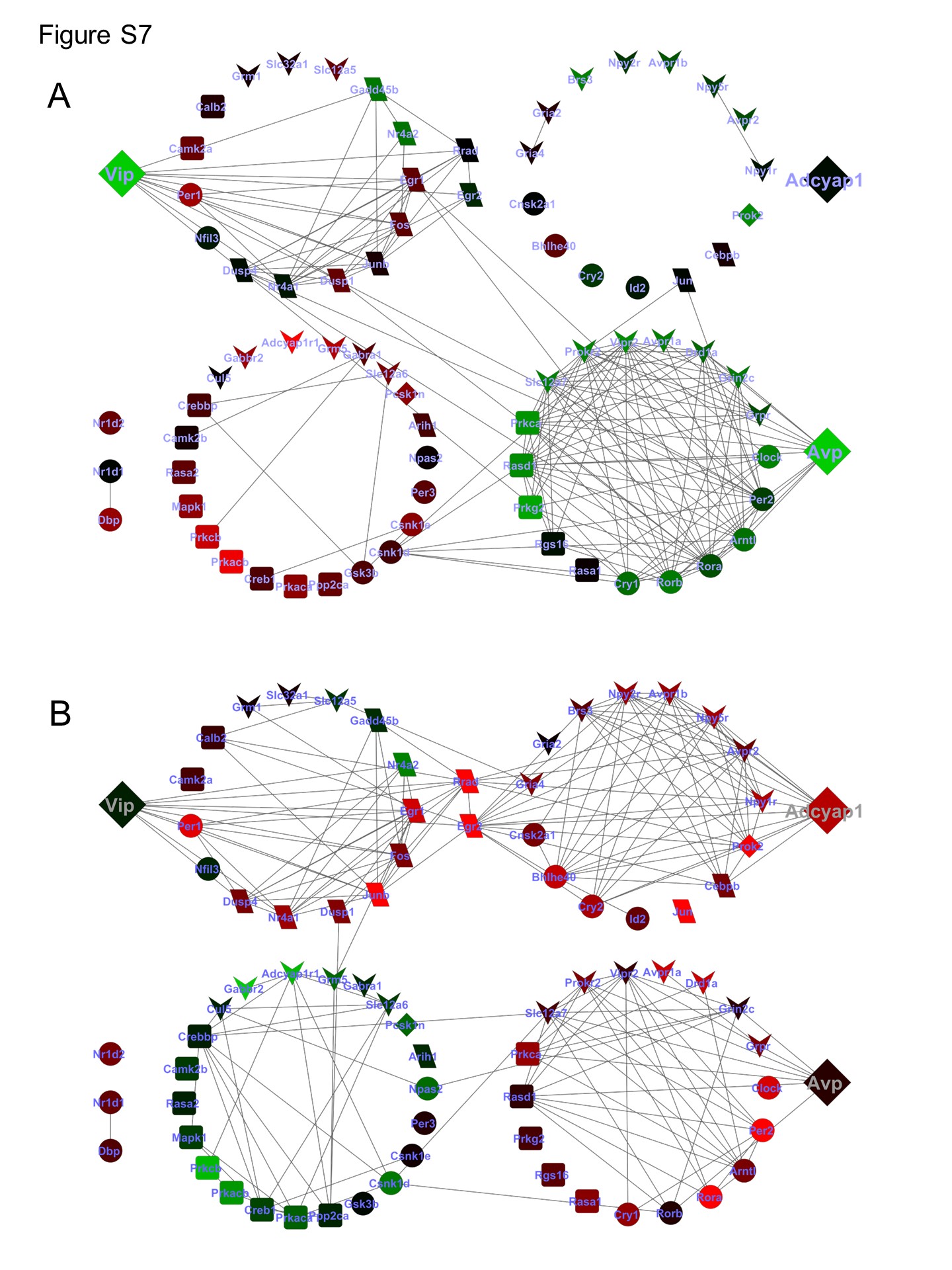

Supplement: Supplementary file 12 [file Image7.jpeg]

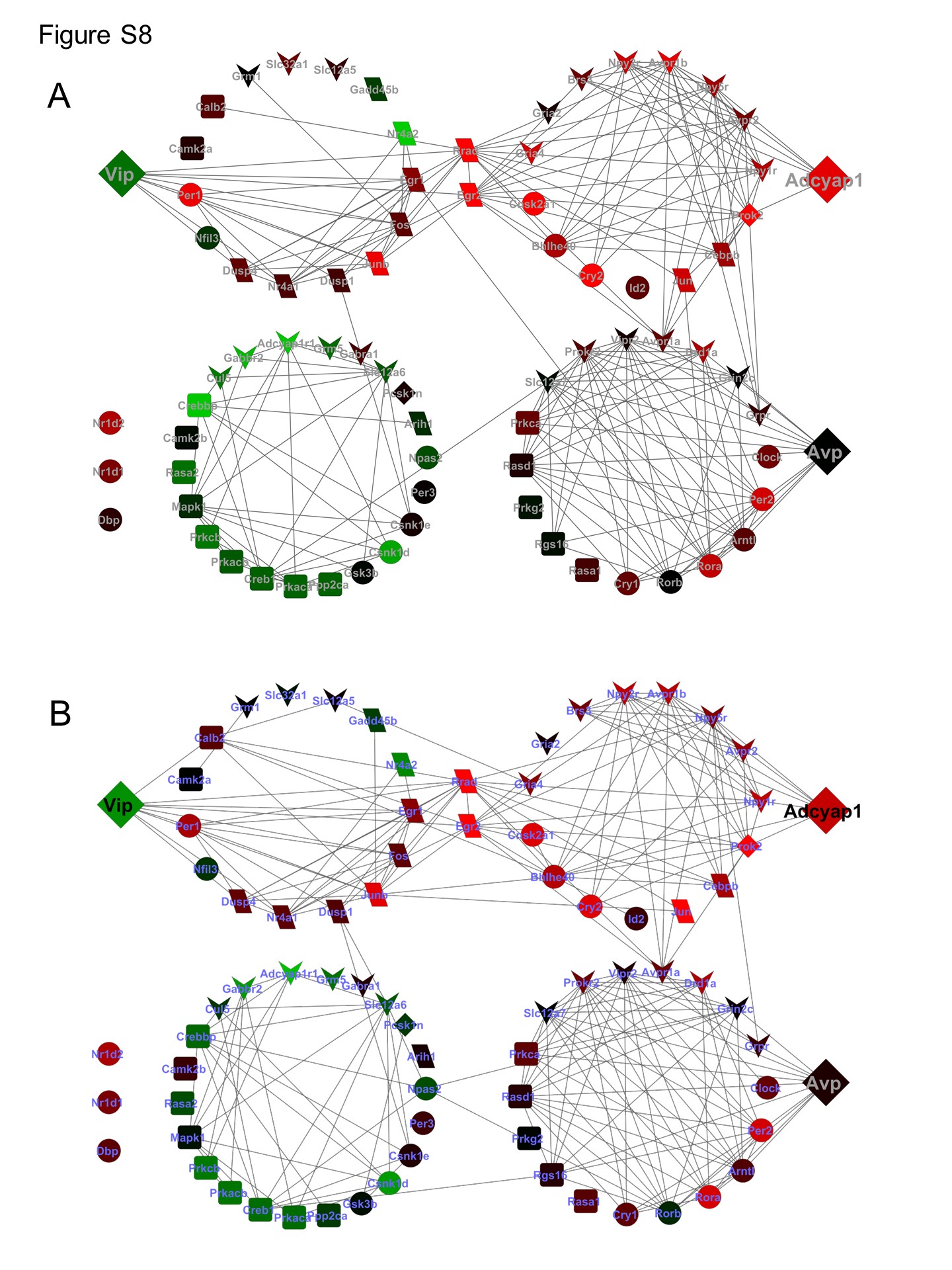

Supplement: Supplementary file 13 [file Image8.jpeg]

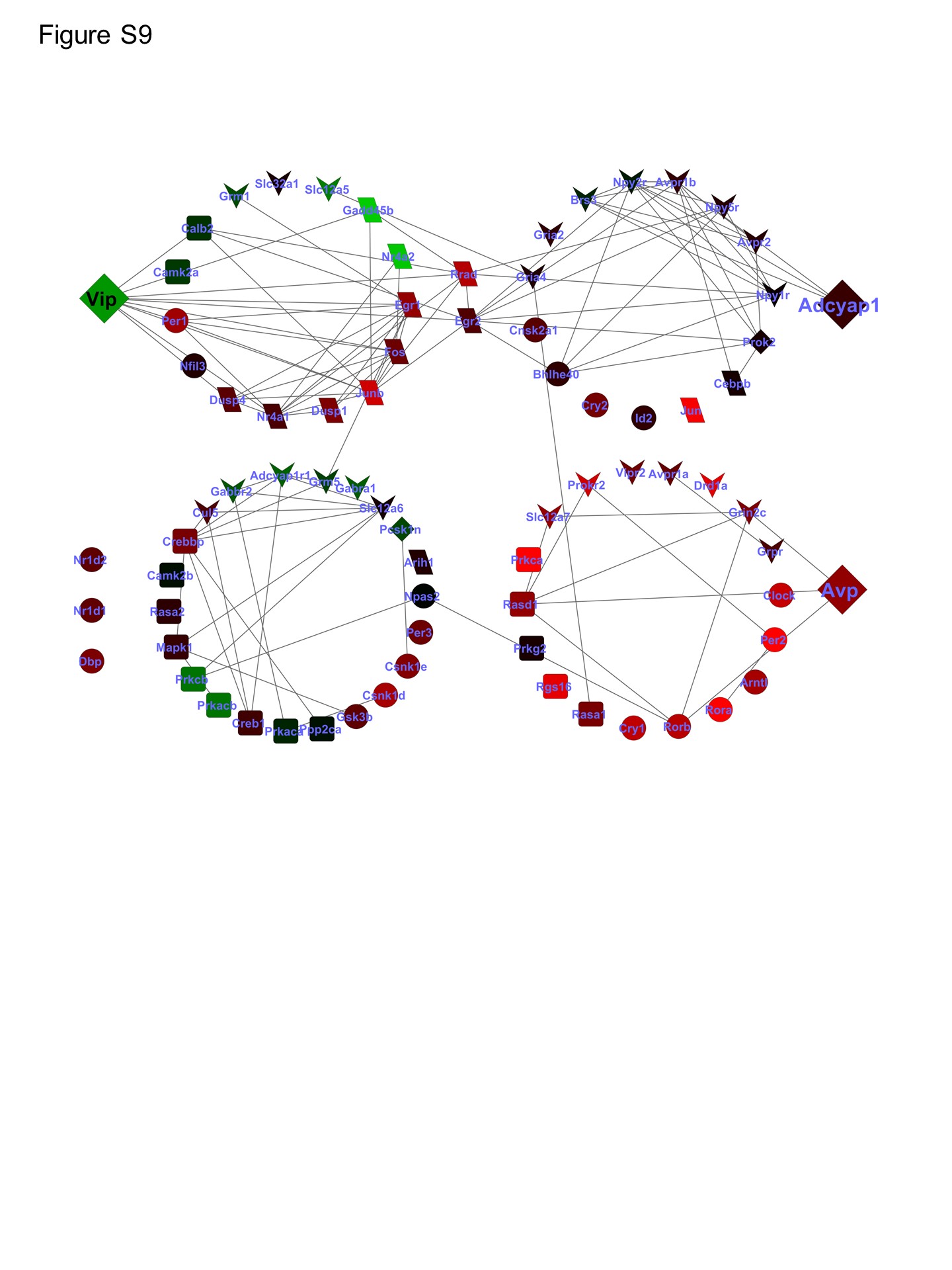

Supplement: Supplementary file 14 [file Image9.jpeg]

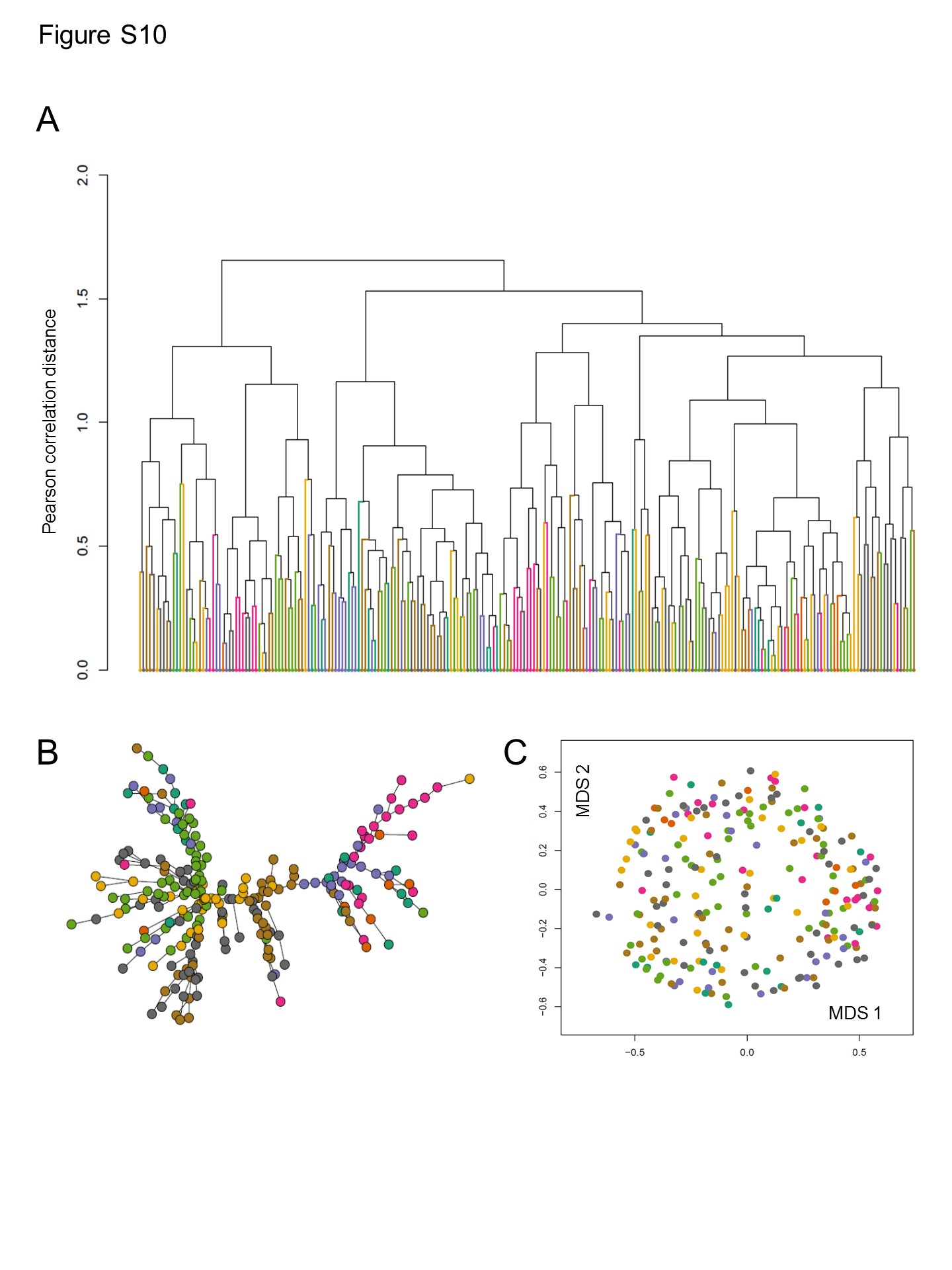

Supplement: Supplementary file 15 [file Image10.jpeg]

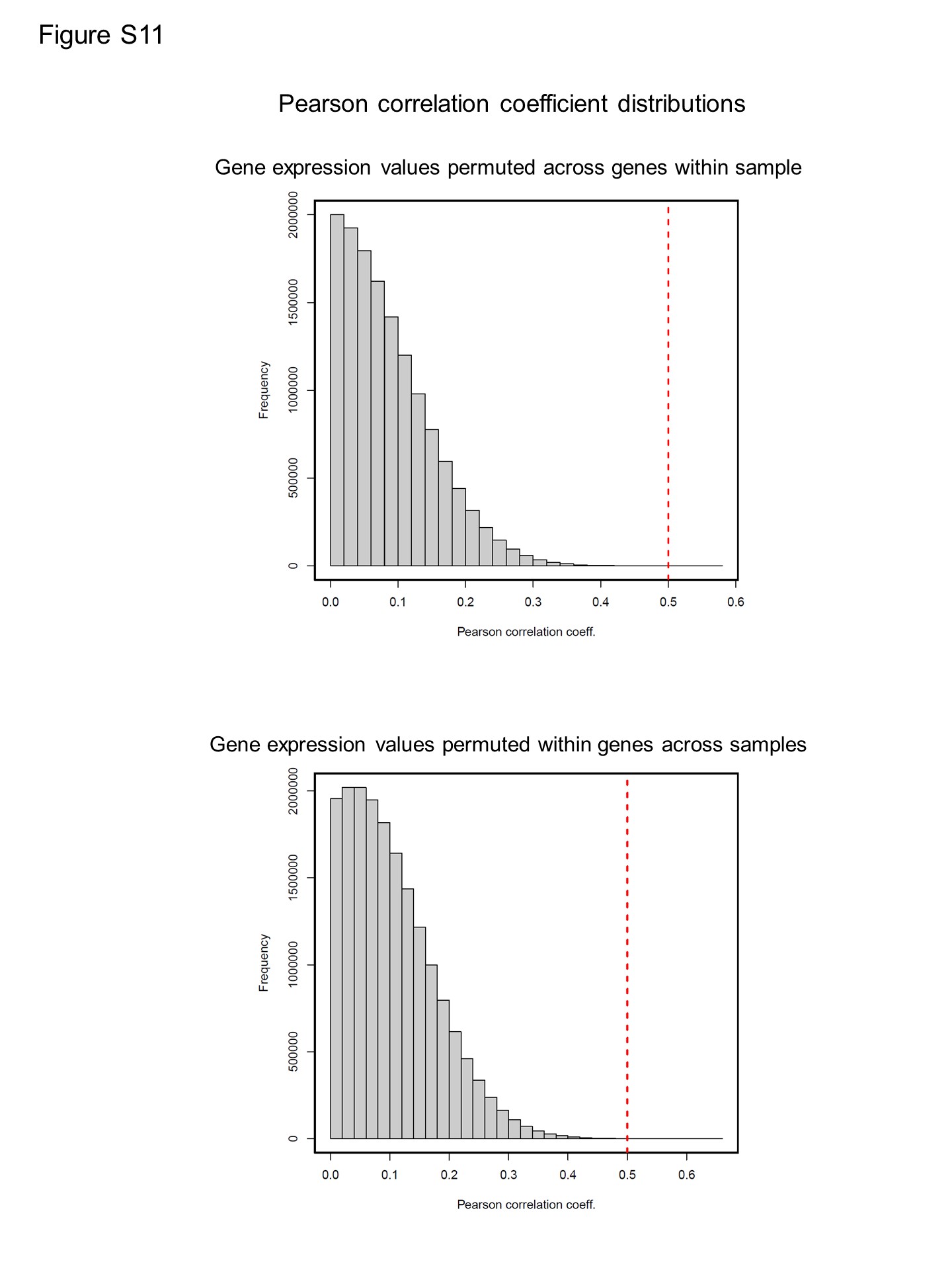

Supplement: Supplementary file 16 [file Image11.jpeg]

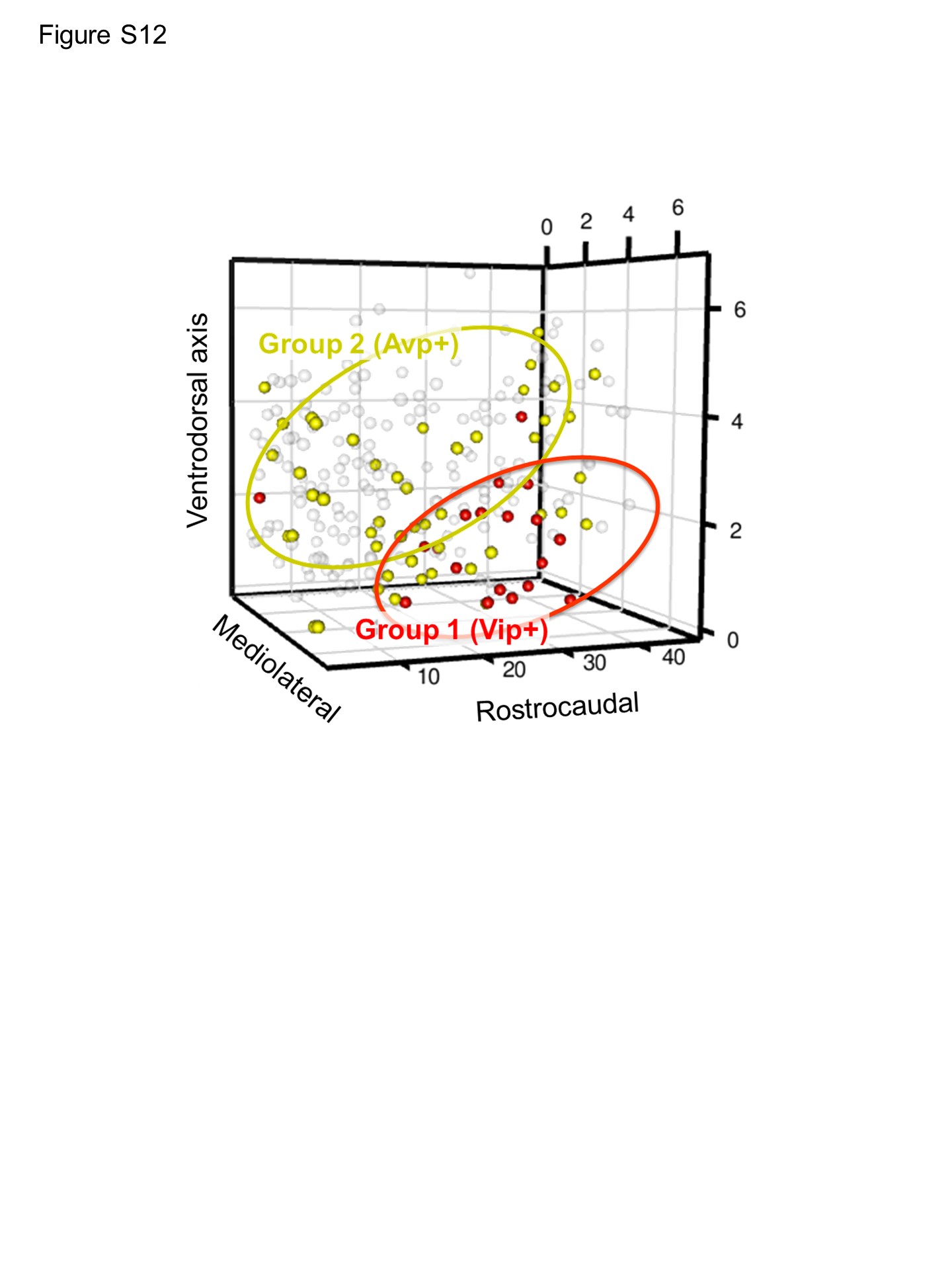

Supplement: Supplementary file 17 [file Image12.jpeg]

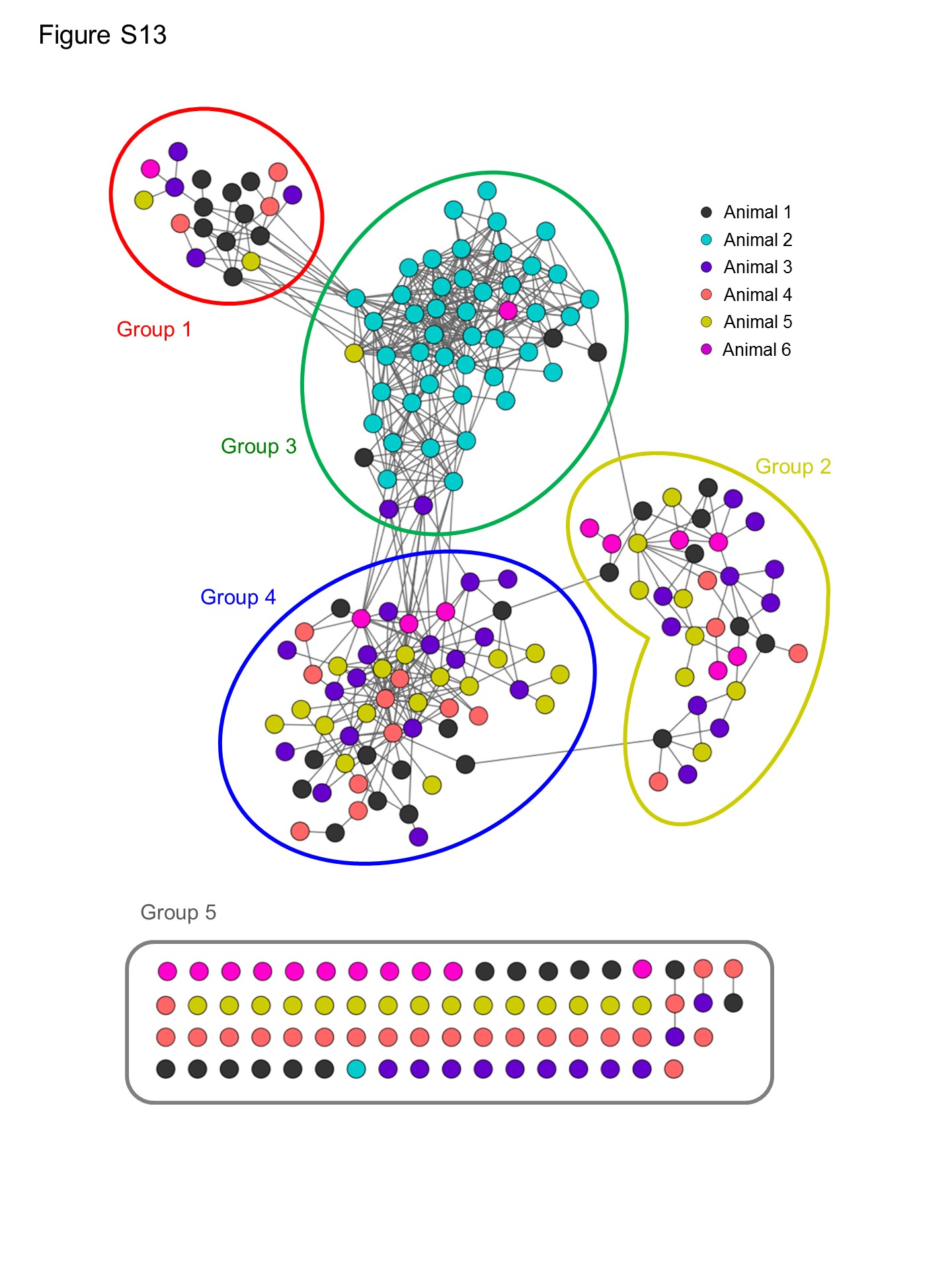

Supplement: Supplementary file 18 [file Image13.jpeg]
